# Enhanced signal spaces assisted media-based modulation in index modulation-based MIMO networks

**DOI:** 10.1038/s41598-025-20541-5

**Published:** 2025-10-21

**Authors:** Jianrong Huang, Bitie Lan, Guowei Li, Zhili Zhou, Fuchun Huang

**Affiliations:** 1 School of Electronic Information and Artificial Intelligence, Wuzhou University, Wuzhou, 543003 China; 2 School of Electrical and Electronic Engineering, Wenzhou University, Wenzhou, 325035 China

**Keywords:** Extension of antenna index vectors, Three dimension (3D), AI set, Squared minimum Euclidean distance (MED), Engineering, Electrical and electronic engineering

## Abstract

Index modulation, which develops the additional information using the antenna index (AI) domain, is a promising modulation technique for next wireless communications. In view of a rich radio scattering environment around transmit antennas (TAs), utilizing the indexes of channel fade realizations, media-based modulation (MBM), which develops the additional information using the channel index (CI) domain, is a recently potential channel modulation and viewed as a potential key technique for enhancing the reliability of communication systems. In this paper, to simultaneously carry the additional information including the AI and CI information bits, the integration of index modulation and MBM is investigated. Firstly of all, the application of the MBM technique to the extended space index modulation (ESIM) system, called as ESIM-MBM, is investigated to improve the spectral efficiency (SE) and error performance of the multiple-input multiple-output with index modulation (MIMO-IM) systems. Specifically, after obtaining one ESIM vector by modulating the real and imaginary parts of one mapped symbol on one or two active TAs using the selected AI vector, according to the number of active TAs, the real and imaginary parts of one mapped symbol are respectively performed Kronecker products by two selected CI vectors with two subparts of CI bits, result in the transmitted MBM vector. Secondly, in order to further extend the size of signal spaces to improve the AI information, with the combination of one or two signal points from QAM and secondary QAM constellations, a new design of enhanced signal spaces with two active TAs assisted the MBM system (ESS-TTAs-MBM) is proposed to enhance the throughput of the communication system. Furthermore, using the maximum likelihood (ML) at the receiver, the comparisons of detection complexities of the proposed ESIM-MBM and ESS-TTAs-MBM with different MBM-based IM schemes are analyzed, and the theoretical average bit error probability (BEP) is also formulated and shown to match well with the Monte-Carlo simulation results at different TAs and SEs in the high SNR region. Finally, a significant improvement in the SE and bit error performance of the proposed schemes is demonstrated with other classic MBM-based IM schemes such as quadrature spatial modulation assisted MBM (QSM-MBM) and quadrature channel modulation (QCM).

## Introduction

In recent years, media-based modulation (MBM)^1^, also termed channel modulation, has emerged as a prominent research focus for next-generation wireless communications. This technique encodes additional information by leveraging distinct channel realizations created through ON/OFF switching of RF mirrors surrounding each transmit antenna (TA). Recent implementations^2^ employ PIN diodes as RF mirrors, achieving switching speeds of 1–-10 ns and stable channel-state reproducibility in static environments. These advancements support data rates exceeding 1 Gbps in practical MBM deployments, though mobility-induced variations necessitate further calibration. Notably, while reconfigurable intelligent surfaces (RIS) have emerged as an alternative channel modulation approach by dynamically manipulating electromagnetic waves, they face inherent challenges including phase errors that degrade channel capacity^3^ and complex optimization of element configurations for energy-spectral efficiency trade-offs^4^. Critically, the MBM technique enhances spectral efficiency (SE) regardless of whether the transmitter employs single or multiple TAs, underscoring its broad applicability.

In the conventional single-input single-output (SISO)/single-input multiple-output (SIMO) systems, there exist only one TA for the transmission of the source information in the transmitter. Therefore, the source information bits are only mapped into one signal constellation point (CP) from the QAM/PSK constellation to be transmitted. Such that, the higher the transmission rate, the higher the modulation order of the employed signal constellation. Consequently, it will seriously deteriorate the bit error rate (BER) performance. In a short, increasing the modulation order is not a good method for improving the transmission rate. To address this shortcoming, the MBM technique^1^ aided the SISO/SIMO systems has been proposed to enhance the SE. In recent years, a amount of works^5–7^ on the SIMO systems with the MBM technique (SIMO-MBM) have been done. In Ref.^5^, utilizing the development of the channel index domain, the MBM technique is directly applied to the communication system with single RF chain for improving the SE. In Ref.^6^, the BER performance is improved for the SIMO-MBM through optimizing the signal constellations. In Ref.^7^, drawing inspiration from the design philosophy of quadrature spatial modulation (QSM)^8^, the channel index domain in the SIMO-MBM system is extended to the in-phase and quadrature dimensions for conveying two components of the mapped signal CP. However, the limitation for the spatial domain with only one TA limits the potential ability to improve the SE.

In terms of multiple transmit antennas, the multiple-input multiple-output (MIMO) system is the extension of the SIMO system. In recent years, as is known to all, the MIMO system has been wisely used in 5G wireless networks due to improving greatly the SE such as the vertical Bell Labs layered space-time (V-BLAST)^9^ scheme. However, it brings the problems of both the inter-channel interference (ICI) and inter-antennas synchronization (IAS). To relax the ICI and IAS, the concept of the spatial modulation (SM) was proposed in Ref.^10^ in 2008, where only one of all TAs is activated to convey one mapped signal CP. On the premise of the SM system, a amount of works on the variants of the MIMO with the index modulation technique have been proposed for improving the SE and the reliability of transmission. In order to enhance the SE of the SM system, the generalized SM (GSM)^11,12^, in which multiple signal symbols from the QAM/PSK constellation are conveyed on multiple active TAs, have been proposed to implement multiplexing gain on the basis of alleviating the ICI and IAS issues. Then, based on the rich-scattering environment around each TA, the MBM technique is applied to the SM and GSM systems, forming the SM-MBM and GSM-MBM systems^13^ for improving the SE by carrying $n_{\textrm{rf}}$ channel index (CI) information bits, which are used to select one out of $2^{n_{\textrm{rf}}}$ mirror activation patterns (MAPs) created by $n_{\textrm{rf}}$ RF mirrors for the transmission of one mapped symbol on the specified channel fade realization in $2^{n_{\textrm{rf}}}$ independent channel fade realizations at each active TA. With the development of index modulation, on the basis of the design idea of both SM and GSM, enhanced SM (ESM)^14^ is proposed to improve the squared minimum Euclidean distance (MED) between the transmit vectors, in which one primary QAM symbol is modulated when only one TA is activated and two secondary QAM symbols are modulated when two TAs are activated. To further exploit the index domain, GSM with multi-index modulation (GSM-MIM)^15^ is proposed to develop the indexes of the key and vector by utilizing two types of signal constellations such as secondary PAM (SPAM) (e.g., 8-SPAM: $\left\{ { \pm 2 \pm 2j, \pm 2 \pm 4j} \right\}$) and QAM constellations. Nonetheless, although the above-mentioned GSM, ESM, GSM-MIM systems improve the performance of wireless communications, their detection complexities at the receiver are increased exponentially with the number of active TAs in comparison with the SM.

On considering of the cost of the detection complexity, Mesleh, et al proposed the quadrature SM (QSM)^8^ in 2016, which is the extension of the SM system in terms of the space domain, i.e., extending the spatial dimension of TAs to the in-phase and quadrature dimensions for modulating the real and imaginary parts of one mapped symbol, respectively. With the development of the in-phase and quadrature dimensions, a amount of works have done, e.g., quadrature index modulation with three dimensional constellation (QIM-TDC)^16^, signed QSM (SQSM)^17^, spatial modulation with spatial constellation (SM-SC)^18^, extended space index modulation (ESIM)^19^ and spatial modulation with jointing permutation, group and antenna indexes (JPGA-ISM)^20^. Specifically, the QIM-TDC is proposed to develop and optimize the three-dimensional (3D) constellation for the index modulation systems with the in-phase and quadrature dimensions. The SQSM further exploits the space dimensions by the limitations of signal constellation point quadrant based on the conventional QAM constellation. With the aid of two signs of “1” and “*j*”, the SM-SC, in addition to carrying the additional information from the antenna index (AI) domain, has developed the symbol group index (GI) for the additional information. Furthermore, with the combination design of two types of signal constellations with two signs of “1” and “*j*”, the ESIM^19^ is proposed to improve the AI information bits with the same the detection complexity as the QSM. On the premise of the SM-SC, the JPGA-ISM is proposed to exploit the permutation index domain. Compared with the above-mentioned GSM, ESM, GSM-MIM systems, although these systems (i.e., QIM-TDC, SQSM, SM-SC and JPGA-ISM) alleviate the ICI and IAS, they have not greater advantage of reducing the complexity of detection in comparisons with the QSM and ESIM systems. From this, the QSM is directly applied to the MBM system, called as quadrature spatial MBM (QSMBM)^5^, for greatly improving the SE of the QSM. In Ref.^21^, with the aid of one reserved TA, quadrature channel modulation-III (QCM-III) is proposed to transmit the real and imaginary parts of the mapped signal symbol without the overlap of the specified transmit channels, respectively. Furthermore, utilizing the design idea of the space-time block code (STBC), the QSM aided MBM (QSM-MBM)^22^ develops the dispersion matrixes for achieving the diversity gain and improving the BER performance. However, the QSM-MBM does not alleviate the complexity of detection and improve the performance at the cost of the time domain.

Considering the shortcomings of the previous works and the advantage of the ESIM, in this article the ESIM aided the MBM technique is investigated to not only exploit more additional information but also remain the merit of the QSM system. The main contributions of the proposed works are summarized as below:

1. On the basis of the rich scattering environment around each TA, the framework of the ESIM with the combination of the MBM (called as ESIM-MBM) is designed for enhancing the throughput and the error performance. Specifically, for modulating the real and imaginary parts (i.e., ${x}^\Re$ and ${x}^\Im$) of one mapped constellation symbol *x* on two specified fade channels for transmission, the design of the ESIM-MBM is divided into two cases: First of all, according to the design of the ESIM, the spatial vector $\textbf{S} \in C^{N_{\textrm{t}} \times 1}$ is obtained. (i). When only one TA is activated by the selected AI vector with the AI bits, the in-phase and quadrature parts (i.e., $\textbf{S}^\Re$ and $\textbf{S}^\Im$) of the spatial vector $\textbf{S}$ are respectively performed Kronecker products by two selected CI vectors with two subparts of CI bits, result in the transmitted MBM vector $\textbf{X} \in C^{N_{\textrm{t}} N_{\textrm{rf}} \times 1}$, where $N_{\textrm{rf}}=2^{n_{\textrm{rf}}}$, $n_{\textrm{rf}}$ is the number of RF mirrors around each TA. (ii). In two activated TAs with the selected AI vector, the obtained spatial vector $\textbf{S}$ is decomposed into $\textbf{S} = s_\Re \cdot \textbf{e}_{\tau _1 } + s_\Im \cdot \textbf{e}_{\tau _2 }$, where $\textbf{e}_{\tau _1 }$ and $\textbf{e}_{\tau _2 }$ denotes the unit vectors with the $\tau _1$-, $\tau _2$-th row non-zero elements, respectively. Then, $s_\Re \cdot \textbf{e}_{\tau _1 }$ and $s_\Im \cdot \textbf{e}_{\tau _2 }$ are respectively performed Kronecker products by two selected CI vectors, result in the MBM vector $\textbf{X}$.
2. In order for the transmission of more AI information to further exploit the spatial domain, enhanced signal spaces with two active TAs assisted the MBM system (ESS-TTAs-MBM) is proposed to further enhance the throughput. Using two signal CPs from the combination of QAM and SQAM constellations (e.g., 4QAM: $\left\{ { \pm 1 \pm j} \right\}$, 4SQAM-I: $\left\{ { \pm 2 \pm 2j} \right\}$, 4SQAM-II: $\left\{ { \pm 2, \pm 2j} \right\}$) for the modulation of two active TAs, thus it can extend $4C^2_{N_{\textrm{t}}}$ types of signal spaces for the additional information with the AI domain. Consequently, the increased AI information bits: $\left\lfloor {\log _2 \left[ {2(C_{N_{\textrm{t}} }^{\textrm{1}} + 4C_{N_{\textrm{t}} }^{\textrm{2}} ) + 4C_{N_{\textrm{t}} }^{\textrm{2}} } \right] } \right\rfloor - \left\lfloor {\log _2 \left[ {2(C_{N_{\textrm{t}} }^{\textrm{1}} + 4C_{N_{\textrm{t}} }^{\textrm{2}} )} \right] } \right\rfloor$ is increased.
3. Based on the design of the proposed ESIM-MBM and ESS-TTAs-MBM systems, the detection complexities with the ML algorithm are analyzed and compared with the classic QSM-MBM, QCM and SM-MBM. Furthermore, the average bit error probability (BEP) with the ML detector for the proposed ESIM-MBM and ESS-TTAs-MBM systems are evaluated. Finally, the verification of simulation experiments with Matlab platform are provided to prove the effectiveness and advantage of the proposed ESIM-MBM and ESS-TTAs-MBM systems in terms of BER performance and throughput in comparisons with other classic schemes such as QSM-MBM, QCM and SM-MBM.

## Extended signal spaces assisted MBM with RF mirrors

### ESIM aided media-based modulation

**Fig. 1:**
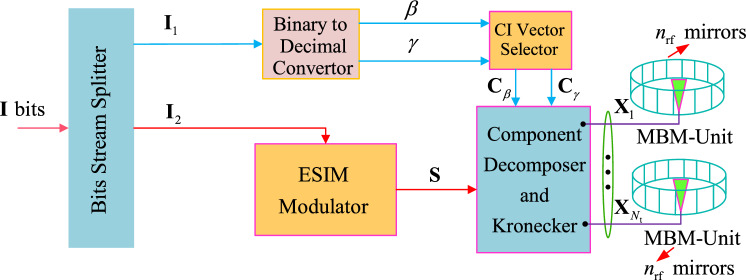
Design model of the proposed ESIM-MBM.

In this subsection, the system model of the ESIM-MBM is introduced, where the transmitter is equipped with $N_{\textrm{t}}$ TAs, where each TA is equipped with one MBM-Unit that is formed by $n_{\textrm{rf}}$ RF mirrors, as is depicted in Fig. 1. Each MBM-Unit employs $n_{\textrm{rf}}$ PIN diode-based RF mirrors^2^, with $n_{\textrm{rf}} \le 4$ to limit switching latency (< 40 ns total). To ensure CI vector stability: The closed-loop control circuits monitor and calibrate RF mirror states in real-time^2^; (ii) Switching slots (e.g., 10 ns) isolate state transitions from data transmission^2^.

In Fig. 1, the input bits of data stream $\textbf{I}$ is divided into two parts: $\textbf{I}_1$ for two specified CI vectors and $\textbf{I}_2$ for the modulated spatial vectors. According to the design principle of the ESIM shown in Ref.^19^, the part of $\textbf{I}_2$ is modulated into one spatial vector $\textbf{S} \in C^{N_{\textrm{t}}\times 1}$ that contains two non-zero components (i.e., $s_\Re$ and $s_\Im$) of the mapped signal CP *s* from the *L*-ary QAM or secondary QAM (SQAM) constellations (seen in Ref.^19^). For the part of $\textbf{I}_1$ bits, by the Binary to Decimal Convertor, it is further divided into two subparts of $\textbf{I}^1_1$ and $\textbf{I}^2_1$, which contain the same CI information bits (i.e., $\textbf{I}^1_1=\textbf{I}^2_1=n_{\textrm{rf}}$), and then are converted into two decimal numbers: $\beta$ and $\gamma$ with two subpart of CI information bits. In the CI vector Selector equipped with a CI vector set $\Upsilon$, $\Upsilon =\{\textbf{C}_1, \dots , \textbf{C}_{N_{\textrm{rf}}}\}$, according to two resulted decimal numbers: $\beta$ and $\gamma$, the CI Vector Selector outputs two CI vectors: $\textbf{C}_\beta$ and $\textbf{C}_\gamma$ to be fed into the Component Decomposer and Kronecker, where these CI vectors are formed by the column vector of a unit matrix $\textbf{I}_{N_{\textrm{rf}}}$ with $N_{\textrm{rf}} \times N_{\textrm{rf}}$ dimensions, $N_{\textrm{rf}}$ denotes the number of possible mirror activation patterns (MAPs) selecting the specified fading channels.

Then, through the module of Component Decomposer and Kronecker, the real $s_\Re$ and imaginary $s_\Im$ of the mapped signal CP *s* in the obtained spatial vector $\textbf{S}$ are respectively modulated on two specified fading channels with two selected CI vectors (i.e., $\textbf{C}_\beta$ and $\textbf{C}_\gamma$). Specifically, in the Component Decomposer and Kronecker, its design rules mainly consider two aspects:

1. Based on the ESIM system, if the selected AI vector is the vector with one non-zero element, then the mapped signal CP *s* is modulated on the activated TA corresponding to the non-zero element, resulting in the spatial vector $\textbf{S}$. In this case, the spatial vector $\textbf{S}$ is first partitioned into the real and imaginary parts: $\textbf{S}_\Re$ and $\textbf{S}_\Im$. Then, based on the kronecker product of matrixes, the two selected CI vectors: $\textbf{C}_\beta$ and $\textbf{C}_\gamma$ respectively modulated by the $\textbf{S}_\Re$ and $\textbf{S}_\Im$, resulting in the transmitted MBM vector $\textbf{X}$, which can be expressed as
  1 $$\begin{aligned} \textbf{X} = \left[ {\begin{array}{*{20}c} {\textbf{X}_1 } \\ \vdots \\ {\textbf{X}_{\alpha } } \\ \vdots \\ {\textbf{X}_{N_{\textrm{t}} } } \\ \end{array}} \right] = \textbf{S}_\Re \otimes \textbf{C}_\beta + j\textbf{S}_\Im \otimes \textbf{C}_\gamma = (s_\Re \cdot \textbf{e}_{\alpha _1} ) \cdot \otimes \textbf{C}_\beta + j(s_\Im \cdot \textbf{e}_{\alpha _1} ) \otimes \textbf{C}_\gamma = \left[ {\begin{array}{*{20}c} \textbf{0} \\ \vdots \\ {s_\Re \otimes \textbf{C}_\beta + js_\Im \otimes \textbf{C}_\gamma } \\ \vdots \\ \textbf{0} \\ \end{array}} \right] , \end{aligned}$$
  where $\otimes$ denotes the operation of the kronecker product, $\textbf{0}$ is the zero vector with the size of $N_{\textrm{rf}} \times 1$ dimensions. ${\alpha }$ is the the index number of the ${\alpha }$-th AI vector in the AI vector set $\Gamma$, which will be introduced in the following, ${\alpha }_1$ is the row number for the non-zero element of the ${\alpha }$-th AI vector, i.e., ${\alpha _1} \in \{1,~2,~\cdots ,~N_{\textrm{t}}\}$, which is the index of the one activated TA. $\textbf{S}_\Re = \chi _\Re \cdot \textbf{e}_{\alpha _1},~\textbf{S}_\Im = \chi _\Im \cdot \textbf{e}_{\alpha _1}$.
2. If the selected AI vector is the vector with two non-zero elements, then the real ${s}_\Re$ and imaginary ${s}_\Im$ of the mapped signal CP *s* are modulated on two active TAs, resulting in the spatial vector $\textbf{S}$. In this case, the spatial vector $\textbf{S}$ is decomposed and given by $\textbf{S} = s_\Re \cdot \textbf{e}_{\alpha _1 } + s_\Im \cdot \textbf{e}_{\alpha _2 }$, where $\textbf{e}_{\alpha _1 }$ and $\textbf{e}_{\alpha _2 }$ denote the unit vectors with the $\alpha _1$-, $\alpha _2$-th row non-zero elements, respectively. In other words, the selected AI vector is expressed by $\textbf{V}=\textbf{e}_{\alpha _1 }+\textbf{e}_{\alpha _2 }$. Then, performing the kronecker product, the transmitted MBM vector $\textbf{X}$ is obtained and given by
  2 $$\begin{aligned} \textbf{X} = \left[ {\begin{array}{*{20}c} {\textbf{X}_1 } \\ \vdots \\ {\textbf{X}_{\alpha _1 } } \\ \vdots \\ {\textbf{X}_{\alpha _2 } } \\ \vdots \\ {\textbf{X}_{N_{\textrm{t}} } } \\ \end{array}} \right] = (s_\Re \cdot \textbf{e}_{\alpha _1 } ) \otimes \textbf{C}_\beta + (s_\Im \cdot \textbf{e}_{\alpha _2 } ) \otimes \textbf{C}_\gamma = \left[ {\begin{array}{*{20}c} \textbf{0} \\ \vdots \\ {s_\Re \cdot \textbf{e}_{\alpha _1 } (\alpha _1 ) \cdot \textbf{C}_\beta } \\ \vdots \\ {s_\Im \cdot \textbf{e}_{\alpha _2 } (\alpha _2 ) \cdot \textbf{C}_\gamma } \\ \vdots \\ \textbf{0} \\ \end{array}} \right] . \end{aligned}$$

where $$\textbf{e}_{\alpha _1 } (\alpha _1 ), \textbf{e}_{\alpha _2 } (\alpha _2 ) \in \{1,~j\}$$, $$\alpha _1 \ne \alpha _2$$.

For the sake of clarity and intuitiveness, Example 1 is provided in the following.

#### Example 1

We set the parameters: $N_{\textrm{t}}=4$, the employed 4-QAM: $\{1+j,-1+j,1-j,-1-j\}$ or 4-SQAM: $\{2+2j,2-2j,-2+2j,-2-2j\}$ , $N_{\textrm{rf}}=2$. Since $N_{\textrm{rf}}=2$, it has the CI vector set $\Upsilon =\{\textbf{C}_1, \dots , \textbf{C}_{4}\}=\{\textbf{e}_1,~\textbf{e}_2,~\textbf{e}_3,~\textbf{e}_4 \}$. In the case of $N_{\textrm{t}}=4$, the AI vector set $\Gamma$ can be expressed as Eq. (9). Assuming that the input bits of data stream is $\textbf{I} = \left[ {\underbrace{1~0~0~1}_{\textbf{I}_1 },\underbrace{\overbrace{1~0}^{\mathrm{For ~one ~CP}}\overbrace{0~0~0~1~0}^{\mathrm{~For ~one ~AI ~vector} }}_{\textbf{I}_2 }} \right]$. Since $I_1 = \left\{ {\underbrace{10}_{I_1^1 }\underbrace{01}_{I_1^2 }} \right\}$, it has $\beta =3$ and $\gamma =2$, so that $\textbf{C}_\beta =\textbf{e}_3$ and $\textbf{C}_\gamma =\textbf{e}_2$. Also, since $\textbf{I}_2=\{1~0, 0~0~0~1~0\}$, it has the mapped signal CP $s=1-j$, and the third AI vector $\textbf{V}_3=[0~0~1~0]^T$ is selected according to the Eq. (9). Then, the spatial vector $\textbf{S}=[0~0~1-j~0]^T$ is obtained by modulating the signal CP $s=1-j$ on the third activated TA with the selected AI vector $\textbf{V}_3=[0~0~1~0]^T$. Furthermore, according to the principle of Eq. (1) and the selected CI vectors: $\textbf{C}_\beta =\textbf{e}_3$ and $\textbf{C}_\gamma =\textbf{e}_2$, the real and imaginary components of the signal CP $s=1-j$ is transmitted over the 3-, 2-th fading channels, respectively. Consequently, the MBM vector is obtained as $\textbf{X}=[0~0~0~0,~0~0~0~0,~0~-j~1~0,~0~0~0~0]^T$. Similarly, if $\textbf{I} = \left[ {1~0~0~1,~1~0,~0~0~1~1~0} \right]$, the AI vector is $\textbf{V}_7=[0~1~1~0]^T$. So that, the real and imaginary components of the signal CP $s=1-j$ is transmitted over the 2-, 3-th activated TAs, respectively. Consequently, the MBM vector can be obtained as $\textbf{X}=[0~0~0~0,~0~0~1~0,~0~-j~0~0,~0~0~0~0]^T$.

### Proposed ESS-TTAs-MBM

**Fig. 2:**
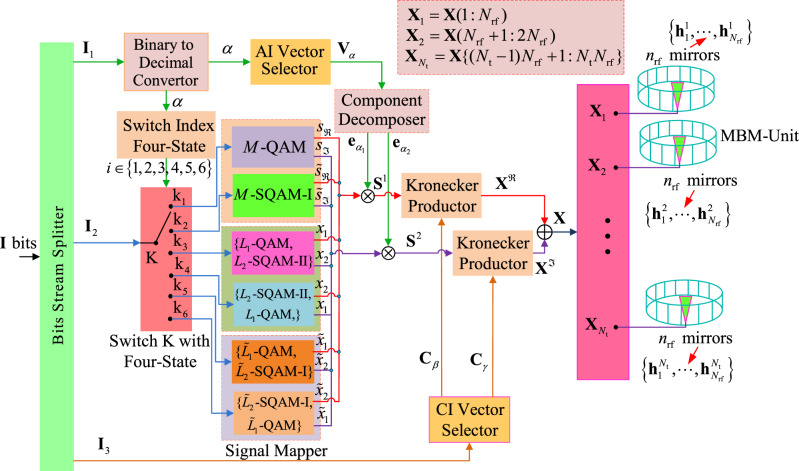
Design model of the proposed ESS-TTAs-MBM.

Although the above-mentioned design of the proposed ESIM-MBM increases the SE of system due to combining with the channel index domain (i.e., the CI information), the AI vectors with one or two active TAs are not been effectively utilized to carry additional information. For instance, assumed $N_{\textrm{t}}=4$. according to the design of the ESIM, it has $2\times (C^1_{4}+4C^2_{4})=56$ AI vectors for carrying the AI bits. However, only $2^{\left\lfloor {{{\textrm{log}} _2 (2\times (C^1_{4}+4C^2_{4}))}} \right\rfloor }=32$ number of AI vectors are legal, the remaining 24 AI vectors are not utilized, where $\left\lfloor {\cdot } \right\rfloor$ represents the floor of operation. In order to make fully use of the un-utilized AI vectors for exploiting the additional information, the design model of the ESS-TTAs-MBM is proposed, as is depicted in Fig. 2.

Figure 2 shows the proposed ESS-TTAs-MBM with $N_{\textrm{t}}$ TAs, the block of data stream bits: $\textbf{I}$ is modulated into one MBM vector $\textbf{X} \in C^{N_{\textrm{rf}}N_{\textrm{t}}\times 1}$ and then transmitted over the specified fading channels with the subblock of CI information bits after being mapped.

Specifically, through the Bits Stream Splitter of Fig. 2, the input block of data stream $\textbf{I}$ is split into three subblocks: ${\textbf{I}_1},~{\textbf{I}_2},~{\textbf{I}_3}$, where ${ {\textbf{I}_1}}=\log _2 N$ bits for the selection of an AI vector from the AI vector set ${\tilde{\Gamma }}$ that will be introduced in the section “Methods”, ${ {\textbf{I}_2}}=\log _2 L$ bits for the mapping of one or two signal CPs from multiple types of signal constellations (e.g., QAM, SQAM-I and SQAM-II), ${{\textbf{I}_3}}=2n_{\textrm{rf}}$ bits for the selection of a CI vector from the CI vector set $\Upsilon$. Note that, *N* is the number of the AI vectors in the set ${\tilde{\Gamma }}$.

For the subblock of ${\textbf{I}_1}$, it is converted into a decimal number $\alpha$ through the Binary to Decimal Convertor. In order to make the specified AI vector modulate one or two specified signal constellations from the QAM, SQAM-I and SQAM-II, the decimal number $\alpha$ is used to not only select a specified AI vector from the AI vector set ${\tilde{\Gamma }}$ but also control the module of Switch Index Four-State (i.e., the outputs has four states: 1, 2, 3, 4), whereupon the decimal number $\alpha$ is fed into two modules of both the AI Vector Selector and Switch Index Four-State. In the AI Vector Selector, with the input of the decimal number $\alpha$, the AI vector $\textbf{V}_\alpha$ is selected out from the AI vector set ${\tilde{\Gamma }}$ that is composed of six subsets: ${\tilde{\Gamma }}_1$, ${\tilde{\Gamma }}_2$, ${\tilde{\Gamma }}_3$, ${\tilde{\Gamma }}_4$, ${\tilde{\Gamma }}_5$, ${\tilde{\Gamma }}_6$, i.e., ${\tilde{\Gamma }}=\{{\tilde{\Gamma }}_1, ~{\tilde{\Gamma }}_2,~{\tilde{\Gamma }}_3,~{\tilde{\Gamma }}_4,~{\tilde{\Gamma }}_5,~{\tilde{\Gamma }}_6\}$, which will be introduced in the section “Extension of the signal spaces”. Then, through the Component Decomposer, the selected AI vector $\textbf{V}_\alpha$ is decomposed into two unit vectors: $\textbf{e}_{\alpha _1}$ and $\textbf{e}_{\alpha _2}$, where the $\alpha _1$-th non-zero element $\textbf{e}_{\alpha _1}(\alpha _1)\in \{1,~j\}$, the $\alpha _2$-th non-zero element $\textbf{e}_{\alpha _2}(\alpha _2)\in \{1,~j\}$.

In the Switch Index Four-State, according to the size of the decimal number $\alpha$, the output has six cases, as follows:

1. If the variable $\alpha$ is less than or equal to $\left| {{\tilde{\Gamma }} _1 } \right|$, the output *i* of the Switch Index Four-State is “1”, i.e., $i=1$.
2. If $\left| {{\tilde{\Gamma }} _1 } \right| +1 \le \alpha \le \left| {{\tilde{\Gamma }} _1 } \right| +\left| {{\tilde{\Gamma }} _2 } \right|$, the output *i* of the Switch Index Four-State is “2”, i.e., $i=2$.
3. If $\left| {{\tilde{\Gamma }} _1 } \right| +\left| {{\tilde{\Gamma }} _2 } \right| +1 \le \alpha \le \left| {{\tilde{\Gamma }} _1 } \right| +\left| {{\tilde{\Gamma }} _2 } \right| +\left| {{\tilde{\Gamma }} _3 } \right|$, the output *i* of the Switch Index Four-State is “3”, i.e., $i=3$.
4. If $\left| {{\tilde{\Gamma }} _1 } \right| +\left| {{\tilde{\Gamma }} _2 } \right| +\left| {{\tilde{\Gamma }} _3 } \right| +1 \le \alpha \le \left| {{\tilde{\Gamma }} _1 } \right| +\left| {{\tilde{\Gamma }} _2 } \right| +\left| {{\tilde{\Gamma }} _3 } \right| +\left| {{\tilde{\Gamma }} _4 } \right|$, the output *i* of the Switch Index Four-State is “4”, i.e., $i=4$.
5. If $\left| {{\tilde{\Gamma }} _1 } \right| +\left| {{\tilde{\Gamma }} _2 } \right| +\left| {{\tilde{\Gamma }} _3 } \right| +\left| {{\tilde{\Gamma }} _4 } \right| +1 \le \alpha \le \left| {{\tilde{\Gamma }} _1 } \right| +\left| {{\tilde{\Gamma }} _2 } \right| +\left| {{\tilde{\Gamma }} _3 } \right| +\left| {{\tilde{\Gamma }} _4 } \right| +\left| {{\tilde{\Gamma }} _5 } \right|$, the output *i* of the Switch Index Four-State is “5”, i.e., $i=5$.
6. If $\left| {{\tilde{\Gamma }} _1 } \right| +\left| {{\tilde{\Gamma }} _2 } \right| +\left| {{\tilde{\Gamma }} _3 } \right| +\left| {{\tilde{\Gamma }} _4 } \right| +\left| {{\tilde{\Gamma }} _5 } \right| +1 \le \alpha \le \left| {{\tilde{\Gamma }} } \right|$, the output *i* of the Switch Index Four-State is “6”, i.e., $i=6$.

Based on the above-mentioned design rules, at the *i*-th case, the Switch K links up the port $\hbox {k}_i$. Then, the subblock of ${ {\textbf{I}_2}}$ is fed into the specified Signal Mapper and then mapped into one signal CP (i.e., *s* from *L*-QAM or ${\tilde{s}}$ from *L*-SQAM-I) or two signal CPs (i.e., $\{x_1,~x_2\}$ from {($L_1$-QAM, $L_2$-QAM-II)} or $\{x_2,~x_1\}$ from {($L_2$-QAM-II, $L_1$-QAM)}, or $\{{{\tilde{x}}}_1,~{{\tilde{x}}}_2\}$ from {(${{\tilde{L}}}_1$-QAM, ${{\tilde{L}}}_2$-QAM-I)} or $\{{{\tilde{x}}}_2,~{{\tilde{x}}}_1\}$ from {(${{\tilde{L}}}_2$-QAM-I, ${{\tilde{L}}}_1$-QAM)}), as shown in Signal Mapper of Fig. 2. In order to make the real and imaginary parts of one mapped signal CP, or two mapped signal CPs be modulated on one or two specified TAs, they are respectively multiplied by two decomposed unit vectors: $\textbf{e}_{\alpha _1}$, $\textbf{e}_{\alpha _2}$ into the spatial vector $\textbf{S}^1$ or $\textbf{S}^2$. More specifically, if the subblock of ${ {\textbf{I}_2}}$ is mapped into the signal CP *s* or ${\tilde{s}}$, the two spatial vectors $\textbf{S}^1$, $\textbf{S}^2$ are obtained by multiplying $s_\Re$/${{\tilde{s}}}_\Re$ and $s_\Im$/${{\tilde{s}}}_\Im$ with $\textbf{e}_{\alpha _1}$, $\textbf{e}_{\alpha _2}$, respectively. Similarly, if the subblock of ${ {\textbf{I}_2}}$ is mapped into two signal CPs from the combination of two signal constellations: {($x_1, x_2$), ($x_2, x_1$),(${{\tilde{x}}}_1, {{\tilde{x}}}_2$),(${{\tilde{x}}}_2, {{\tilde{x}}}_1$)}, the two spatial vectors $\textbf{S}^1$, $\textbf{S}^2$ are obtained by multiplying the selected CPs combination (e.g., ($x_1, x_2$)) with $\textbf{e}_{\alpha _1}$, $\textbf{e}_{\alpha _2}$, respectively. In order to intuitively clarify the expression of two spatial vectors: $\textbf{S}^1$, $\textbf{S}^2$, the mathematical expressions of two spatial vectors: $\textbf{S}^1$, $\textbf{S}^2$ may be given by

3 $$\begin{aligned} \textbf{S}^1 = \left\{ \begin{array}{ll} s_\Re \cdot \textbf{e}_{\alpha _1 } ~~~~s_\Re ~\textrm{from }~L\mathrm{- QAM} \\ {\tilde{s}}_\Re \cdot \textbf{e}_{\alpha _1 } ~~~~{\tilde{s}}_\Re ~\textrm{from }~L\mathrm{- SQAM - I} \\ x_1 \cdot \textbf{e}_{\alpha _1 } ~~~~x_1 ~\textrm{from }~L_{\textrm{1}} \mathrm{- QAM} \\ x_2 \cdot \textbf{e}_{\alpha _1 } ~~~~x_2 ~\textrm{from }~L_{\textrm{2}} \mathrm{- SQAM - II} \\ {\tilde{x}}_1 \cdot \textbf{e}_{\alpha _1 } ~~~~{\tilde{x}}_1~ \textrm{from }~{\tilde{L}}_{\textrm{1}} \mathrm{- QAM} \\ {\tilde{x}}_2 \cdot \textbf{e}_{\alpha _1 } ~~~~{\tilde{x}}_2~ \textrm{from }~{\tilde{L}}_{\textrm{2}} \mathrm{- SQAM - I} \\ \end{array} \right. ,\textbf{S}^2 = \left\{ \begin{array}{ll} s_\Im \cdot \textbf{e}_{\alpha _2 } ~~~~s_\Im ~ \textrm{from }~L\mathrm{- QAM} \\ {\tilde{s}}_\Im \cdot \textbf{e}_{\alpha _2 }~~~~{\tilde{s}}_\Im ~\textrm{from }~L\mathrm{- SQAM - I} \\ x_2 \cdot \textbf{e}_{\alpha _2 } ~~~~x_2~ \textrm{from }~L_{\textrm{2}}~ \mathrm{- SQAM - II} \\ x_1 \cdot \textbf{e}_{\alpha _2 } ~~~~x_1~ \textrm{from }~L_{\textrm{1}}~ \mathrm{- QAM} \\ {\tilde{x}}_2 \cdot \textbf{e}_{\alpha _2 } ~~~~{\tilde{x}}_2 ~\textrm{from }~{\tilde{L}}_{\textrm{2}} ~\mathrm{- SQAM - I} \\ {\tilde{x}}_1 \cdot \textbf{e}_{\alpha _2 } ~~~~{\tilde{x}}_1~ \textrm{from }~{\tilde{L}}_{\textrm{1}} ~\mathrm{- QAM} \\ \end{array} \right. . \end{aligned}$$

where $$L=L_1 \times L_2={{{\tilde{L}}_1} \times {{\tilde{L}}_2}}$$.

For the subblock of ${\textbf{I}_3}$, it is divided into two subblocks of $\textbf{I}^1_3=n_{\textrm{rf}}$ and $\textbf{I}^2_3=n_{\textrm{rf}}$, which are respectively used to select two CI vectors: $\textbf{C}_\beta$ and $\textbf{C}_\gamma$ from the CI vector set $\Upsilon =\{\textbf{C}, \cdots , \textbf{C}_{N_{\textrm{rf}}}\}$ that is one unit matrix with $N_{\textrm{rf}} \times N_{\textrm{rf}}$ dimensions, where $\beta$ and $\gamma$ are the decimal numbers of two subblocks of $n_{\textrm{rf}}, n_{\textrm{rf}}$, respectively. In order to transmit the real and imaginary parts of one mapped signal CP or two mapped signal CPs on two specified fading channels, two Kronecker Products are employed and controlled by two selected CI vectors: $\textbf{C}_\beta$ and $\textbf{C}_\gamma$. Consequently, in the two Kronecker Products, the real/imaginary part ${\textbf{X}^\Re }/\textbf{X}^\Im$ of the MBM vector $\textbf{X}$ are obtained by performing the kronecker product of both the spatial vector ${\textbf{S}^1}/\textbf{S}^2$ and the selected CI vector ${\textbf{C}_\beta }/\textbf{C}_\gamma$. Thus, the MBM vector can be obtained by adding the real part $\textbf{X}^\Re$ to the imaginary part $\textbf{X}^\Im$, as follows:

4 $$\begin{aligned} \begin{aligned}&\textbf{X} = \textbf{X}^\Re + \textbf{X}^\Im \\&= \left\{ \begin{array}{l} \textbf{S}^{\textrm{1}} \otimes \textbf{C}_\beta + \textbf{S}^{\textrm{2}} \otimes \textbf{C}_\gamma = (s_\Re \cdot \textbf{e}_{\alpha _1 } ) \otimes \textbf{C}_\beta {}+ (s_\Im \cdot \textbf{e}_{\alpha _2 } ) \otimes \textbf{C}_\gamma ~~\mathrm{for~ }~~L\mathrm{- QAM} \\ \textbf{S}^{\textrm{1}} \otimes \textbf{C}_\beta + \textbf{S}^{\textrm{2}} \otimes \textbf{C}_\gamma = ({\tilde{s}}_\Re \cdot \textbf{e}_{\alpha _1 } ) \otimes \textbf{C}_\beta + ({\tilde{s}}_\Im \cdot \textbf{e}_{\alpha _2 } ) \otimes \textbf{C}_\gamma ~~ \textrm{for }~~~L -\mathrm{SQAM-I} \\ \textbf{S}^{\textrm{1}} \otimes \textbf{C}_\beta + \textbf{S}^{\textrm{2}} \otimes \textbf{C}_\gamma = (x_1 \cdot \textbf{e}_{\alpha _1 } ) \otimes \textbf{C}_\beta + (x_2 \cdot \textbf{e}_{\alpha _2 } ) \otimes \textbf{C}_\gamma ~~~ \textrm{for } \{ ~L_{\textrm{1}} \mathrm{- QAM, }L_{\textrm{2}} \mathrm{- SQAM - II } \} \\ \textbf{S}^{\textrm{1}} \otimes \textbf{C}_\beta + \textbf{S}^{\textrm{2}} \otimes \textbf{C}_\gamma = (x_2 \cdot \textbf{e}_{\alpha _1 } ) \otimes \textbf{C}_\beta {+ (}x_1 \cdot \textbf{e}_{\alpha _2 } ) \otimes \textbf{C}_\gamma ~~ ~\textrm{for }\{ L_{\textrm{2}} \mathrm{- SQAM - II, }~L_{\textrm{1}} \mathrm{- QAM} \} \\ \textbf{S}^{\textrm{1}} \otimes \textbf{C}_\beta + \textbf{S}^{\textrm{2}} \otimes \textbf{C}_\gamma = ({\tilde{x}}_1 \cdot \textbf{e}_{\alpha _1 } ) \otimes \textbf{C}_\beta {+ (}{\tilde{x}}_2 \cdot \textbf{e}_{\alpha _2 } ) \otimes \textbf{C}_\gamma ~~~ \textrm{for }~~~\{ {\tilde{L}}_{\textrm{1}} \mathrm{- QAM, }~{\tilde{L}}_{\textrm{2}} {{\mathrm{- SQAM - I}}}\} \\ \textbf{S}^{\textrm{1}} \otimes \textbf{C}_\beta + \textbf{S}^{\textrm{2}} \otimes \textbf{C}_\gamma = ({\tilde{x}}_2 \cdot \textbf{e}_{\alpha _1 } ) \otimes \textbf{C}_\beta {+ (}{\tilde{x}}_1 \cdot \textbf{e}_{\alpha _2 } ) \otimes \textbf{C}_\gamma ~~~ \textrm{for }~~~\{ {\tilde{L}}_{\textrm{2}} \mathrm{- SQAM - I, }~{\tilde{L}}_{\textrm{1}} {\mathrm{- QAM}}\} \\ \end{array} \right. \\ \end{aligned}. \end{aligned}$$

Note that, for the cases of modulating both *L*-QAM and *L*-SQAM-I, $\alpha _1=\alpha _2$ in Eq. (4).

For the sake of clarity and intuitiveness, $\textbf{Example 2}$ is provided in the following.

#### Example 2

We set the parameters: $N_{\textrm{t}}=4$, the employed signal CP(s) may be 4-QAM: $\{1+j,1-j,-1+j,-1-j\}$ or 4-SQAM-I: $\{2+2j,2-2j,-2+2j,-2-2j\}$ or both 2-QAM: $\{1+j,-1-j\}$ and 2-SQAM-II: $\{2, 2j\}$, $N_{\textrm{rf}}=2$. Since $N_{\textrm{rf}}=2$, it has the CI vector set $\Upsilon =\{\textbf{C}_1, \dots , \textbf{C}_{4}\}=\{\textbf{e}_1,~\textbf{e}_2,~\textbf{e}_3,~\textbf{e}_4 \}$. According to the design of Eq. (10), if $N_{\textrm{t}}=4$, the AI vector set ${\tilde{\Gamma }}$ can be obtained as Eq. (5). Assuming that the input bits of data stream is $\textbf{I} = \left[ {\underbrace{\overbrace{1~1~1~1~0~~1}^{\mathrm{For~ one ~AI ~vector}}}_{I_1 }\underbrace{\overbrace{0~1}^{\mathrm{~For ~two~ CPs}}}_{I_2 }\underbrace{1~1~0~1}_{I_3 }} \right]$. Since $I_3 = \left\{ {\underbrace{1~1}_{I_3^1 }\underbrace{0~1}_{I_3^2 }} \right\}$, it has $\beta =4$ and $\gamma =2$, so that $\textbf{C}_\beta =\textbf{e}_4$ and $\textbf{C}_\gamma =\textbf{e}_2$. Also, since $\textbf{I}_2=\{1~1~1~1~0~1\}$, it has $\alpha =62$. Thus, according to the Eq. (5), the $I_2$ bits are used to modulate both 2-QAM and 2-SQAM-II constellations, and then two mapped signal CPs: $(x_1, x_2)=(1+j,2j)$ may be obtained. Also, due to $\alpha =62$, the AI vector $\textbf{V}_{62}=[0~0 ~1~1]^T=\textbf{e}_3+\textbf{e}_4$ is selected according to the Eq. (5). Furthermore, with the selected AI vector $\textbf{V}_{62}$, the spatial vector $\textbf{S}^1=[0~0~1+j~0]^T$ and $\textbf{S}^2=[0~0~0~2j]^T$ can be obtained by modulating the signal CP $x_1=1+j$ and $x_2=2j$ on two specified TAs, respectively. Finally, with the aid of two CI vectors: $\textbf{C}_\beta =\textbf{e}_4$ and $\textbf{C}_\gamma =\textbf{e}_2$ in the two Keonecker Products, the MBM vector is obtained as $\textbf{X}=[~0~0~0~0,~0~0~0~0,0~0~0~1+j,~0~2j~0~0]^T$.

5 $$\begin{aligned} {\tilde{\Gamma }} = \left\{ \begin{array}{l} \overbrace{\underbrace{\begin{array}{*{20}c} {1 + j} & 0 & 0 & 0 \\ 0 & {1 + j} & 0 & 0 \\ 0 & 0 & {1 + j} & 0 \\ 0 & 0 & 0 & {1 + j} \\ \end{array}}_{C_4^{\textrm{1}} ~\mathrm{AI ~vectors}}\textrm{,}\underbrace{\begin{array}{*{20}c} \textrm{1} & \textrm{1} & 0 & 0 & 1 & 0 \\ 0 & 0 & \textrm{1} & \textrm{1} & 1 & 0 \\ \textrm{1} & 0 & \textrm{1} & 0 & 0 & 1 \\ 0 & \textrm{1} & 0 & \textrm{1} & 0 & 1 \\ \end{array}}_{C_4^{\textrm{2}}~ \mathrm{AI ~vectors}},\underbrace{\begin{array}{*{20}c} 1 & 1 & 0 & 0 & 1 & 0 \\ 0 & 0 & 1 & 1 & j & 0 \\ j & 0 & j & 0 & 0 & 1 \\ 0 & j & 0 & j & 0 & j \\ \end{array}}_{C_4^{\textrm{2}}~ \mathrm{AI ~vectors}},\underbrace{\begin{array}{*{20}c} j & j & 0 & 0 & j & 0 \\ 0 & 0 & j & j & 1 & 0 \\ 1 & 0 & 1 & 0 & 0 & j \\ 0 & 1 & 0 & 1 & 0 & 1 \\ \end{array},}_{C_4^{\textrm{2}} ~\mathrm{AI ~vectors}}}^{~\mathrm{Modulating ~the ~mapped ~QAM ~CPs}} \\ \underbrace{\overbrace{\begin{array}{*{20}c} j & j & 0 & 0 & j & 0 \\ 0 & 0 & j & j & j & 0 \\ j & 0 & j & 0 & 0 & j \\ 0 & j & 0 & j & 0 & j \\ \end{array}}^{~\mathrm{Modulating ~the ~mapped ~QAM ~CPs}}}_{C_4^{\textrm{2}} ~\mathrm{AI ~vectors}}\overbrace{\underbrace{\begin{array}{*{20}c} {1 + j} & 0 & 0 & 0 \\ 0 & {1 + j} & 0 & 0 \\ 0 & 0 & {1 + j} & 0 \\ 0 & 0 & 0 & {1 + j} \\ \end{array}}_{C_4^{\textrm{1}}~ \mathrm{AI ~vectors}}\textrm{,}\underbrace{\begin{array}{*{20}c} \textrm{1} & \textrm{1} & 0 & 0 & 1 & 0 \\ 0 & 0 & \textrm{1} & \textrm{1} & 1 & 0 \\ \textrm{1} & 0 & \textrm{1} & 0 & 0 & 1 \\ 0 & \textrm{1} & 0 & \textrm{1} & 0 & 1 \\ \end{array}}_{C_4^{\textrm{2}}~ \mathrm{AI ~vectors}},\underbrace{\begin{array}{*{20}c} 1 & 1 & 0 & 0 & 1 & 0 \\ 0 & 0 & 1 & 1 & j & 0 \\ j & 0 & j & 0 & 0 & 1 \\ 0 & j & 0 & j & 0 & j \\ \end{array},}_{C_4^{\textrm{2}} ~\mathrm{AI ~vectors}}}^{~\mathrm{Modulating ~the~ mapped ~QAM - I ~CPs}} \\ \\ \overbrace{\underbrace{\begin{array}{*{20}c} j & j & 0 & 0 & j & 0 \\ 0 & 0 & j & j & 1 & 0 \\ 1 & 0 & 1 & 0 & 0 & j \\ 0 & 1 & 0 & 1 & 0 & 1 \\ \end{array},}_{C_4^{\textrm{2}} \mathrm{AI~ vectors}}\underbrace{\begin{array}{*{20}c} j & j & 0 & 0 & j & 0 \\ 0 & 0 & j & j & j & 0 \\ j & 0 & j & 0 & 0 & j \\ 0 & j & 0 & j & 0 & j \\ \end{array}}_{C_4^{\textrm{2}}~ \mathrm{AI ~vectors}}}^{\mathrm{~Modulating ~the~ mapped ~SQAM - I ~CPs}}\underbrace{\begin{array}{*{20}c} 1 & 1 & 0 & 0 & 1 & 0 & 1 & 0 \\ 0 & 0 & 1 & 1 & 1 & 0 & 1 & 1 \\ 1 & 0 & 1 & 0 & 0 & 1 & 0 & 1 \\ 0 & 1 & 0 & 1 & 0 & 1 & 0 & 0 \\ \end{array}}_{\mathrm{8 ~AI ~vectors ~for ~the ~combination~ of ~the ~QAM ~and ~SQAM - II }} \\ \end{array} \right\} \end{aligned}$$

### Receiver

At the $n_{\mathrm t}$-th MBM-Unit ($1 \le n_{\mathrm t} \le N_{\mathrm t}$) in the right side of both Figs. 1 and 2, $n_{\textrm{rf}}$ RF mirrors can form $N_{\textrm{rf}}=2^{n_{\textrm{rf}}}$ possible channel states: ${{\textbf { h}}}_1^{n_{\mathrm t} }, \cdots ,{{\textbf { h}}}_{N_{\textrm{rf}} }^{n_{\mathrm t} } \in C^{N_{\mathrm r}\times 1}$ for constructing the channel states matrix: ${{\textbf { H}}}^{n_{\mathrm t} }$, which can be expressed by ${{\textbf { H}}}^{n_{\mathrm t} }=[{{\textbf { h}}}_1^{n_{\mathrm t} }, {{\textbf { h}}}_2^{n_{\mathrm t} },\cdots ,{{\textbf { h}}}_{N_{\mathrm t} }^{n_{\mathrm t} }]$. Accordingly, for $N_{\mathrm t}$ TAs whose each is equipped with $n_{\textrm{rf}}$ RF mirrors, the fading channel matrix ${\textbf { H}}$ can be expressed by

6 $$\begin{aligned} \begin{array}{l} {{\textbf { H}}} = [{{\textbf { H}}}^1 ,{{\textbf { H}}}^2 , \cdots ,{{\textbf { H}}}^{N_{\mathrm t} } ] \\ = [\underbrace{{{\textbf { h}}}_1^1 , \cdots ,{{\textbf { h}}}_{N_{{\textrm{rf}}} }^1 }_{1{ - { \textrm{st}} ~\textrm{ TA}}},\underbrace{{{\textbf { h}}}_1^2 , \cdots ,{{\textbf { h}}}_{N_{{\textrm{rf}}} }^2 }_{{\mathrm 2 - { \textrm{nd}}~ \textrm{TA}}}, \cdots ,\underbrace{{{\textbf { h}}}_1^{N_{\mathrm t} } , \cdots ,{{\textbf { h}}}_{N_{{\textrm{rf}}} }^{N_{\mathrm t} } }_{N_{\mathrm t} { - {\textrm{th}}~ \textrm{TA}}}] \\ \end{array}, \end{aligned}$$

where ${{\textbf { H}}} \in C^{N_{\mathrm r}\times {N_{\mathrm t} N_{\textrm{rf}} }}$, ${{\textbf { h}}}^{n_{\mathrm t}}_\lambda \in C^{{N_{\mathrm r} }\times 1} ( \lambda \in \{1, \cdots , N_{\textrm{rf}}\})$ is the $\lambda$-th fading channel between the $n_{\mathrm t}$-th TA and $N_{\mathrm r}$ receive antennas, whose item obeys the Rayleigh fading with zero mean and “1” variance, i.e., following *CN*(0, 1).

Based on above-mentioned design of $N_{\mathrm t}$ MBM-Units, the *k*-th sub-MBM vector $\textbf{X}_k$ is transmitted over the specified fading channel at the *k*-th MBM-Unit. Thus, for $N_{\textrm{t}}$ sub-MBM vectors: $\textbf{X}_1,~\textbf{X}_2,~\cdots ,~\textbf{X}_{N_{\textrm{t}}}$ at $N_{\textrm{t}}$ MBM-Units, the received signal vector $\textbf{Y}$ can be expressed as

7 $$\begin{aligned} \begin{array}{l} \textbf{Y} = \frac{1}{\mu }{\textbf{H}} \cdot {\textbf{X}} + {\textbf{n}} \\ \\ = \frac{1}{\mu }[\textbf{H}^1 ,\textbf{H}^2 , \cdots ,\textbf{H}^{N_{\textrm{t}} } ] \cdot \left[ {\textbf{X}^1 , \cdots , \cdots ,\textbf{X}^{N_{\textrm{t}} } } \right] ^T + \textbf{n} \\ \\ = \frac{1}{\mu }[\underbrace{\textbf{h}_1^1 , \cdots ,\textbf{h}_{N_{r\textrm{f}} }^1 }_{1\mathrm{- st ~TA}},\underbrace{\textbf{h}_1^2 , \cdots ,\textbf{h}_{N_{r\textrm{f}} }^2 }_{\mathrm{2 - nd~ TA}}, \cdots ,\underbrace{\textbf{h}_1^{N_{\textrm{t}} } , \cdots ,\textbf{h}_{N_{r\textrm{f}} }^{N_{\textrm{t}} } }_{N_{\textrm{t}} \mathrm{- th~ TA}}] \cdot \left[ {\textbf{X}^1 , \cdots , \cdots ,\textbf{X}^{N_{\textrm{t}} } } \right] ^T + \textbf{n} \\ \\ \mathrm{= }\left\{ \begin{array}{l} \frac{1}{\mu }\left( {s_\Re \cdot \textbf{h}_\alpha ^\beta + js_\Im \cdot \textbf{h}_\alpha ^\gamma } \right) + \textbf{n}\begin{array}{*{20}c} {} & {} \\ \\ \end{array}\mathrm{With ~one ~active~ TA} \\ \\ \frac{1}{\mu }\left( {s_\Re \cdot \textbf{e}_{\alpha _1 } (\alpha _1 ) \cdot \textbf{h}_{\alpha _1 }^\beta + s_\Im \cdot \textbf{e}_{\alpha _2 } (\alpha _2 ) \cdot \textbf{h}_{\alpha _2 }^\gamma } \right) + \textbf{n}\begin{array}{*{20}c} {} & {} \\ \end{array}\mathrm{With~ two ~active ~TAs} \\ \end{array} \right. \\ \end{array}' \end{aligned}$$

where $\mu$ is the factor of normalizing the MBM vector.

With the assumption of the perfectly known channel state information (CSI), on the basis of the above design, the received vector symbol $\textbf{y}$ in Eq. (7) is jointly detected with the index $\alpha$ of the selected AI vector from the AI vector set, the mapped constellation symbol $Z,~Z\in \{s,~{\tilde{s}}, (x_1, x_2), (x_2, x_1),({{\tilde{x}}}_1, {{\tilde{x}}}_2),({{\tilde{x}}}_2, {{\tilde{x}}}_1) \}$ and the indexes $\beta ,~\gamma$ of two CI vectors for retrieving the original information bits of *I*. Consequently, with using the ML algorithm, the mathematical expression of detective algorithm for the proposed systems can be given by

8 $$\begin{aligned} \begin{array}{l} \left[ {{\hat{\alpha }} ,{\hat{Z}},{\hat{\beta }} ,{\hat{\gamma }} } \right] = \arg \mathop {\min }\limits _{\alpha ,Z,\beta ,\gamma } \left\| {\textbf{y} - \textbf{H} \cdot \frac{1}{\mu } \textbf{X}} \right\| ^2 \\ = \left\{ \begin{array}{l} \arg \mathop {\min }\limits _{\alpha ,Z,\beta ,\gamma } \left\| {\textbf{y} - \frac{1}{\mu }\left( {s_\Re \cdot \textbf{h}_\alpha ^\beta + js_\Im \cdot \textbf{h}_\alpha ^\gamma } \right) } \right\| ^2 \begin{array}{*{20}c} {} & {} \\ \end{array}\mathrm{With ~one ~active~ TA} \\ \arg \mathop {\min }\limits _{\alpha ,Z,\beta ,\gamma } \left\| {\textbf{y} - \frac{1}{\mu }\left( {s_\Re \cdot \textbf{e}_{\alpha _1 } (\alpha _1 ) \cdot \textbf{h}_{\alpha _1 }^\beta + s_\Im \cdot \textbf{e}_{\alpha _2 } (\alpha _2 ) \cdot \textbf{h}_{\alpha _2 }^\gamma } \right) } \right\| ^2 \begin{array}{*{20}c} {} & {} \\ \end{array}\mathrm{With ~two ~active ~TAs} \\ \end{array} \right. \\ \end{array} \end{aligned}$$

where $\left\| \cdot \right\| ^2$ is the operation of Frobenius norm, ${\hat{\alpha }}$ is the estimate of the index of the selected AI vector from the AI vector set $\Gamma$ or ${\tilde{\Gamma }}$. ${\hat{Z}}$ is the estimate of the mapped one or two signal CPs. ${\hat{\beta }},{\hat{\gamma }}$ are the estimates of two selected CI vectors.

## Methods

### Review of the signal spaces in ESIM system

In order to betterly understand the improved ESIM vector, we first introduce the principle of generating the ESIM vector.

On the premise of keeping the merit of the squared MED $d_{\min ,\textbf{X}}^2 = 2$, utilizing two types of signal constellations (i.e., QAM and secondary QAM) and the symbol index groups (e.g., four symbol index groups: $\left\{ {\left( {1,1} \right) ,\left( {1,i} \right) ,\left( {i,1} \right) ,\left( {i,i} \right) } \right\}$), the signal spaces for the ESIM are expanded to exploit the additional spatial index bits in comparison with the QSM, i.e., Modulating two types of signal CPs with these AI vectors used to activate one or two TAs and with the aid of four symbol index groups: $\left\{ {\left( {1,1} \right) ,\left( {1,i} \right) ,\left( {i,1} \right) ,\left( {i,i} \right) } \right\}$, Thus it has $C^1_{N\textrm{t}}+4C^2_{N\textrm{t}}$ AI vectors for modulating the QAM symbols and $C^1_{N\textrm{t}}+4C^2_{N\textrm{t}}$ AI vectors for the SQAM symbols. However, the number of transmitted AI bits needs to be an integer multiple of one bit. In other words, it needs to satisfy the power of two. According to the design principle of the ESIM, the AI vector set $\Upsilon$ used to modulate two types of QAM and SQAM CP symbols contains $N_1=2^{\left\lfloor {{{\textrm{log}} _2 (2\times (C^1_{N\textrm{t}}+4C^2_{N\textrm{t}}))}} \right\rfloor }$ number of AI vectors, i.e., Only $N_1=2^{\left\lfloor {{{\textrm{log}} _2 (2\times (C^1_{N\textrm{t}}+4C^2_{N\textrm{t}}))}} \right\rfloor }$ out of $N= 2\times (C^1_{N\textrm{t}}+4C^2_{N\textrm{t}})$ AI vectors are used to construct the AI vector set $\Gamma$ for carrying the AI information bits, the remaining $N-N_1$ number of AI vectors are discarded. Consequently, multiple index combinations of active antenna(s) are not fully utilized, resulting in wastage of active TA(s) combination resources, especially in the MIMO systems with a large number of TAs. For a clearer understanding, an example is given. Assumed that $N\textrm{t}=4$, the AI vector set denoted by $\Gamma$, which is used to modulate the mapped QAM and SQAM CPs, are as follows:

9 $$\begin{aligned} \Gamma = \left\{ \begin{array}{l} \overbrace{\underbrace{\begin{array}{*{20}c} 1 & 0 & 0 & 0 \\ 0 & 1 & 0 & 0 \\ 0 & 0 & 1 & 0 \\ 0 & 0 & 0 & 1 \\ \end{array}}_{C_4^{\textrm{1}}~ \mathrm{AI~ vectors}}\textrm{,}\underbrace{\begin{array}{*{20}c} \textrm{1} & \textrm{1} & 0 & 0 & 1 & 0 \\ 0 & 0 & \textrm{1} & \textrm{1} & 1 & 0 \\ \textrm{1} & 0 & \textrm{1} & 0 & 0 & 1 \\ 0 & \textrm{1} & 0 & \textrm{1} & 0 & 1 \\ \end{array}}_{C_4^{\textrm{2}}~ \mathrm{AI ~vectors}},\underbrace{\begin{array}{*{20}c} 1 & 1 & 0 & 0 & 1 & 0 \\ 0 & 0 & 1 & 1 & 1 & 0 \\ 1 & 0 & 1 & 0 & 0 & 1 \\ 0 & 1 & 0 & 1 & 0 & 1 \\ \end{array}}_{C_4^{\textrm{2}}~ \mathrm{AI ~vectors}},\underbrace{\begin{array}{*{20}c} 1 & 1 & 0 & 0 & 1 & 0 \\ 0 & 0 & 1 & 1 & 1 & 0 \\ 1 & 0 & 1 & 0 & 0 & 1 \\ 0 & 1 & 0 & 1 & 0 & 1 \\ \end{array}}_{C_4^{\textrm{2}} ~\mathrm{AI ~vectors}},\underbrace{\begin{array}{*{20}c} 1 & 1 & 0 & 0 & 1 & 0 \\ 0 & 0 & 1 & 1 & 1 & 0 \\ 1 & 0 & 1 & 0 & 0 & 1 \\ 0 & 1 & 0 & 1 & 0 & 1 \\ \end{array}}_{C_4^{\textrm{2}} ~\mathrm{AI ~vectors}}}^{\mathrm{Modulating ~the~ mapped ~QAM~ CPs}} \\ \\ \overbrace{\underbrace{\begin{array}{*{20}c} 1 & 0 & 0 & 0 \\ 0 & 1 & 0 & 0 \\ 0 & 0 & 1 & 0 \\ 0 & 0 & 0 & 1 \\ \end{array}}_{C_4^{\textrm{1}} ~\mathrm{AI~ vectors}}\textrm{,}\underbrace{\underbrace{\begin{array}{*{20}c} \textrm{1} & \textrm{1} & 0 & 0 & 1 & 0 \\ 0 & 0 & \textrm{1} & \textrm{1} & 1 & 0 \\ \textrm{1} & 0 & \textrm{1} & 0 & 0 & 1 \\ 0 & \textrm{1} & 0 & \textrm{1} & 0 & 1 \\ \end{array}}_{C_4^{\textrm{2}} ~\mathrm{AI~ vectors}},\underbrace{\begin{array}{*{20}c} 1 & 1 & 0 & 0 & 1 & 0 \\ 0 & 0 & 1 & 1 & 1 & 0 \\ 1 & 0 & 1 & 0 & 0 & 1 \\ 0 & 1 & 0 & 1 & 0 & 1 \\ \end{array}}_{C_4^{\textrm{2}}~ \mathrm{AI ~vectors}},\underbrace{\begin{array}{*{20}c} 1 & 1 & 0 & 0 & 1 & 0 \\ 0 & 0 & 1 & 1 & 1 & 0 \\ 1 & 0 & 1 & 0 & 0 & 1 \\ 0 & 1 & 0 & 1 & 0 & 1 \\ \end{array}}_{C_4^{\textrm{2}}~ \mathrm{AI~ vectors}},\underbrace{\begin{array}{*{20}c} 1 & 1 & 0 & 0 & 1 & 0 \\ 0 & 0 & 1 & 1 & 1 & 0 \\ 1 & 0 & 1 & 0 & 0 & 1 \\ 0 & 1 & 0 & 1 & 0 & 1 \\ \end{array}}_{C_4^{\textrm{2}}~ \mathrm{AI~ vectors}}}_{4C_4^{\textrm{2}}~ \mathrm{discarded ~AI~ vectors}}}^{\mathrm{Modulating ~the ~mapped~ SQAM~ CPs}} \\ \end{array} \right\} \end{aligned}$$

From the Eq. (9), the first $C^1_{4}+4C^2_{4}$ AI vectors are to modulate the mapped QAM CPs and the remaining AI vectors are to modulate the mapped SQAM-I CPs, i.e., there exists $N= 2\times (C^1_{4}+4C^2_{4})=56$ AI vectors for carrying the AI bits. Due to the number of AI vectors need to satisfy the power of two, only $N_1=2^{\left\lfloor {{{\textrm{log}} _2 (2\times (C^1_{4}+4C^2_{4}))}} \right\rfloor }=32$ number of AI vectors constructing the AI vector $\Gamma$ are legal for carrying five AI information bits, the remaining $N_2=N-N_1=24$ AI vectors are discarded. Thus, with $N_{\textrm{t}}$ number of TAs, it will have $2 \times (C_{N_{\textrm{t}} }^{\textrm{1}} + 4C_{N_{\textrm{t}} }^{\textrm{2}} ) - 2^{\left\lfloor {{\textrm{log}}_2 \left( {2 \times (C_{N_{\textrm{t}} }^{\textrm{1}} + 4C_{N_{\textrm{t}} }^{\textrm{2}} )} \right) } \right\rfloor }$ AI vectors to be discarded. From this, it can be seen that the number of discarded AI vectors is greatly increased with the increasing of the number of TAs. Hence, the design schemes on being capable of making fully use of the discarded AI vectors are worth further developing.

Based on the above-mentioned issue and analysis, we draw inspiration from the design concept of SSD-MCVA^23^, which joints multiple constellations and variable active antennas selection for signal spaces design. To further expand the signal spaces of the ESIM, we fully utilize the discarded AI vectors.

### Extension of the signal spaces

In the ESS-TTAs-MBM system, the design criteria of expanding the signal spaces is based the following two points:

1. To further exploit more additional information bits from the index combinations of one or two active antennas, keep merit of the squared MED $d_{\min ,\textbf{X}}^2 = 2$, the combinations of two different types of signal constellations are employed.
2. In the design process, in order to integrate with the MBM system, the AI vectors with activating one TA need to be further modified.

According to these design criterias, with the consideration of the average energy of each signal space, the QAM, SQAM-I and SQAM-II constellations (e.g., 2QAM: $\{1+j, -1-j\}$, 2SQAM-II: $\{2, -2\}$) are employed. On the premise of the AI vector set $\Gamma$ in the ESIM-MBM system, the design rule of all AI vectors used to construct the extended AI vector set ${\tilde{\Gamma }}$ for the ESS-TTAs-MBM may be described, as follows:

1. Similar to the ESIM system, being used to modulate one mapped QAM CP symbol, it has $C^1_{N\textrm{t}}+4C^2_{N\textrm{t}}$ AI vectors, which are formed by one or two active TAs with the aid of four symbol index groups: $\left\{ {\left( {1,1} \right) ,\left( {1,i} \right) ,\left( {i,1} \right) ,\left( {i,i} \right) } \right\}$. Among them, the two non-zero elements for $4C^2_{N\textrm{t}}$ AI vectors being used to activate two TAs can be set as ${\left( {1,1} \right) ,\left( {1,i} \right) ,\left( {i,1} \right) ,\left( {i,i} \right) }$. Nonetheless, the one non-zero element for $C^1_{N\textrm{t}}$ AI vectors being used to activate only one TA is different from the ESIM system and set as $1+j$, the real and imaginary parts of which are used to modulate the real and and imaginary parts of the mapped QAM CP symbol, respectively.
2. Similar to the above case, being used to modulate one SQAM-I CP symbol, there exists $C^1_{N\textrm{t}}+4C^2_{N\textrm{t}}$ AI vectors, which are formed by one or two active TAs with the aid of four symbol index groups. Among them, two non-zero elements in $4C^2_{N\textrm{t}}$ AI vectors are also set as ${\left( {1,1} \right) ,\left( {1,i} \right) ,\left( {i,1} \right) ,\left( {i,i} \right) }$. And, the one non-zero element for $C^1_{N\textrm{t}}$ AI vectors being used to activate only one TA is set as $1+j$, whose real and and imaginary parts is used to modulate the real and and imaginary parts of the mapped SQAM-I CP symbol, respectively.
3. On the premise of the above two cases, to extend the signal spaces, the permutating method is utilized for the combination of two types of signal constellations. That is to say, one QAM symbol and one SQAM-II symbol (or both QAM and SQAM-I) are permutated in order and modulated respectively on two active TAs. Specifically, for the AI vectors using to activate two active TAs, it can be expressed as $\textbf{V} = \textbf{e}_{\xi _1 } + \textbf{e}_{\xi _2 }$, where $\textbf{e}_{\xi _1 }$ and $\textbf{e}_{\xi _2 }$ denote two unit vectors being used to activate the $\xi _1$-, $\xi _2$-th TAs, respectively. On the one hand, when the $\textbf{e}_{\xi _1 }$ and $\textbf{e}_{\xi _2 }$ are respectively used to modulate the QAM and SQAM-II (or both QAM and SQAM-I) CPs, there exists $C^2_{N\textrm{t}}$ AI vectors. On the other hand, when the $\textbf{e}_{\xi _1 }$ and $\textbf{e}_{\xi _2 }$ are respectively used to modulate the SQAM-II and QAM (or both SQAM-I and QAM) CPs, it also has $C^2_{N\textrm{t}}$ AI vectors. Thus, with the permutating method, it can increase $4C^2_{N\textrm{t}}$ AI vectors for carrying the additional spatial index information bits.

Consequently, it has $2\times (C^1_{N\textrm{t}}+4C^2_{N\textrm{t}})+4C^2_{N\textrm{t}}$ number of AI vectors to construct the AI vector set ${\tilde{\Gamma }}$ for forming more signal spaces. Due to the additional information bits carried need to be an integer multiple of one bit, $2^{\left\lfloor {{{\textrm{log}} _2 (2\times (C^1_{N\textrm{t}}+4C^2_{N\textrm{t}})+4C^2_{N\textrm{t}})}} \right\rfloor }$ out of $2\times (C^1_{N\textrm{t}}+4C^2_{N\textrm{t}})+4C^2_{N\textrm{t}}$ AI vectors are employed for constructing the extended AI vector set ${\tilde{\Gamma }}$. It can be seen that, compared with the ESIM system, the proposed ESS-TTAs-MBM not only improves the utilization efficiency of the AI vectors but also enhances the transmission of the additional information. For instance, assuming $N\textrm{t}=4$, according to the above design, it has 64 out of $2\times (C^1_{4}+4C^2_{4})+4C^2_{4}=80$ AI vectors for constructing the AI vector set ${\tilde{\Gamma }}$, as depicted in Eq. (5). Consequently, only 16 AI vectors are discarded.

It is worth noting that, with the consideration of the squared MED ($d_{\textbf{X},\min }^2 = \frac{2}{{E_{\textrm{av}}^{\textrm{MBM}} }}$, ${E_{\textrm{av}}^{\textrm{MBM}} }$ denotes the average energy of each MBM vector) between the MBM vectors, the combination of the QAM with the SQAM-II is first employed in comparison with the combination of the QAM and the SQAM-I. Therefore, based on the above-mentioned design and analysis, all AI vectors for constructing the AI vector set ${\tilde{\Gamma }}$ can be designed as

10 $$\begin{aligned} \Delta = \left\{ \begin{array}{l} \overbrace{\underbrace{\begin{array}{*{20}c} {1 + j} & 0 & \cdots & 0 \\ 0 & {1 + j} & \vdots & \vdots \\ \vdots & 0 & \ddots & 0 \\ \vdots & \vdots & \vdots & \vdots \\ 0 & 0 & \cdots & {1 + j} \\ \end{array}}_{C_{N_{\textrm{t}} }^{\textrm{1}} ~\mathrm{AI ~vectors}}\textrm{,}\underbrace{\begin{array}{*{20}c} 1 & 1 & \cdots & 0 \\ 1 & 0 & \vdots & \vdots \\ 0 & 1 & \ddots & 0 \\ \vdots & \vdots & \vdots & 1 \\ 0 & 0 & \cdots & 1 \\ \end{array}}_{C_{N_{\textrm{t}} }^{\textrm{2}}~ \mathrm{AI~ vectors}},\underbrace{\begin{array}{*{20}c} 1 & 1 & \cdots & 0 \\ j & 0 & \vdots & \vdots \\ 0 & j & \ddots & 0 \\ \vdots & \vdots & \vdots & 1 \\ 0 & 0 & \cdots & j \\ \end{array}}_{C_{N_{\textrm{t}} }^{\textrm{2}} ~\mathrm{AI ~vectors}},\underbrace{\begin{array}{*{20}c} j & j & \cdots & 0 \\ 1 & 0 & \vdots & \vdots \\ 0 & 1 & \ddots & 0 \\ \vdots & \vdots & \vdots & j \\ 0 & 0 & \cdots & 1 \\ \end{array}}_{C_{N_{\textrm{t}} }^{\textrm{2}} ~\mathrm{AI ~vectors}},\underbrace{\begin{array}{*{20}c} j & j & \cdots & 0 \\ j & 0 & \vdots & \vdots \\ 0 & j & \ddots & 0 \\ \vdots & \vdots & \vdots & j \\ 0 & 0 & \cdots & j \\ \end{array}}_{C_{N_{\textrm{t}} }^{\textrm{2}} ~\mathrm{AI ~vectors}}}^{~\mathrm{Modulating ~the mapped~ QAM ~CPs}} \\ \\ \overbrace{\underbrace{\begin{array}{*{20}c} {1 + j} & 0 & \cdots & 0 \\ 0 & {1 + j} & \vdots & \vdots \\ \vdots & 0 & \ddots & 0 \\ \vdots & \vdots & \vdots & \vdots \\ 0 & 0 & \cdots & {1 + j} \\ \end{array}}_{C_{N_{\textrm{t}} }^{\textrm{1}} ~\mathrm{AI ~vectors}}\textrm{,}\underbrace{\begin{array}{*{20}c} 1 & 1 & \cdots & 0 \\ 1 & 0 & \vdots & \vdots \\ 0 & 1 & \ddots & 0 \\ \vdots & \vdots & \vdots & 1 \\ 0 & 0 & \cdots & 1 \\ \end{array}}_{C_{N_{\textrm{t}} }^{\textrm{2}}~ \mathrm{AI ~vectors}},\underbrace{\begin{array}{*{20}c} 1 & 1 & \cdots & 0 \\ j & 0 & \vdots & \vdots \\ 0 & j & \ddots & 0 \\ \vdots & \vdots & \vdots & 1 \\ 0 & 0 & \cdots & j \\ \end{array}}_{C_{N_{\textrm{t}} }^{\textrm{2}} ~\mathrm{AI ~vectors}},\underbrace{\begin{array}{*{20}c} j & j & \cdots & 0 \\ 1 & 0 & \vdots & \vdots \\ 0 & 1 & \ddots & 0 \\ \vdots & \vdots & \vdots & j \\ 0 & 0 & \cdots & 1 \\ \end{array}}_{C_{N_{\textrm{t}} }^{\textrm{2}}~ \mathrm{AI ~vectors}},\underbrace{\begin{array}{*{20}c} j & j & \cdots & 0 \\ j & 0 & \vdots & \vdots \\ 0 & j & \ddots & 0 \\ \vdots & \vdots & \vdots & j \\ 0 & 0 & \cdots & j \\ \end{array}}_{C_{N_{\textrm{t}} }^{\textrm{2}} ~\mathrm{AI ~vectors}}}^{\mathrm{~Modulating ~the ~mapped~ SQAM - I~ CPs}} \\ \overbrace{\underbrace{\begin{array}{*{20}c} 1 & 1 & \cdots & 0 \\ 1 & 0 & \vdots & \vdots \\ 0 & 1 & \ddots & 0 \\ \vdots & \vdots & \vdots & 1 \\ 0 & 0 & \cdots & 1 \\ \end{array}}_{C_{N_{\textrm{t}} }^{\textrm{2}} ~\mathrm{AI~ vectors}},\underbrace{\begin{array}{*{20}c} 1 & 1 & \cdots & 0 \\ 1 & 0 & \vdots & \vdots \\ 0 & 1 & \ddots & 0 \\ \vdots & \vdots & \vdots & 1 \\ 0 & 0 & \cdots & 1 \\ \end{array}}_{C_{N_{\textrm{t}} }^{\textrm{2}} \textrm{AI vectors}}}^{\mathrm{the~ combination of ~the ~QAM ~and~ SQAM - II ~CPs}},\overbrace{\underbrace{\begin{array}{*{20}c} 1 & 1 & \cdots & 0 \\ 1 & 0 & \vdots & \vdots \\ 0 & 1 & \ddots & 0 \\ \vdots & \vdots & \vdots & 1 \\ 0 & 0 & \cdots & 1 \\ \end{array}}_{C_{N_{\textrm{t}} }^{\textrm{2}} ~\mathrm{AI ~vectors}},\underbrace{\begin{array}{*{20}c} 1 & 1 & \cdots & 0 \\ 1 & 0 & \vdots & \vdots \\ 0 & 1 & \ddots & 0 \\ \vdots & \vdots & \vdots & 1 \\ 0 & 0 & \cdots & 1 \\ \end{array}}_{C_{N_{\textrm{t}} }^{\textrm{2}}~ \mathrm{AI ~vectors}},}^{\mathrm{the~ combination ~of~ the~ QAM ~and ~SQAM - I~ CPs}} \\ \end{array} \right\} \end{aligned}$$

From the Eq. (10), the first $2^{\left\lfloor {{{\textrm{log}} _2 (2\times (C^1_{N\textrm{t}}+4C^2_{N\textrm{t}})+4C^2_{N\textrm{t}})}} \right\rfloor }$ number of AI vectors are selected to construct the AI vector set ${\tilde{\Gamma }} = \left\{ {\textbf{V}_1, \cdots ,\textbf{V}_{ 2^{\left\lfloor {{{\textrm{log}} _2 (2\times (C^1_{N\textrm{t}}+4C^2_{N\textrm{t}})+4C^2_{N\textrm{t}})}} \right\rfloor }} } \right\}$.

## Performance analysis

### Spectral efficiency

In this work, the SEs of the two proposed schemes is composed of three parts: the CI bits, the AI vector bits and the mapping bits.

(1). The SE for the ESIM-MBM

According to the design principle of ESIM-MBM and Eq. (9), the maximum SE of the ESIM-MBM is mathematically expressed as

11 $$\begin{aligned} \textrm{SE}_{\textrm{max}}^{\mathrm{ESIM - MBM}} = \textrm{log}_2 \left( {\underbrace{N_1 }_{\mathrm{AI ~vector ~bits}} \times \underbrace{2^{2n_{\textrm{rf}} } }_{\mathrm{CI~ bits}} \times \underbrace{L}_{\mathrm{CP ~index~ bits}}} \right) ~~~~~({\textrm{bits}}/{\textrm{s}}/{\textrm{Hz}}), \end{aligned}$$

where $N_1 = 2^{\left\lfloor {\log _2 2 \cdot (C_{N_{\textrm{t}} }^1 + 4C_{N_{\textrm{t}} }^2 )} \right\rfloor }$ represents the number of valid AI vectors, *L* is the modulation order.

Furthermore, the ESS-TTAs-MBM system achieves enhanced SE by exploiting all available spatial resources through dual-constellation modulation and complete AI vector utilization. Its maximum SE is derived as:

12 $$\begin{aligned} \textrm{SE}_{\textrm{max}}^{\mathrm{ESS - TTAs - MBM}} = \textrm{log}_2 \left( {\underbrace{\left| {{\tilde{\Gamma }} } \right| }_{\mathrm{AI ~vector~ bits}} \times \underbrace{2^{2n_{\textrm{rf}} } }_{\mathrm{CI~ bits}} \times \underbrace{L}_{\mathrm{CP~ index ~bits}}} \right) ~~~~~({\textrm{bits}}/{\textrm{s}}/{\textrm{Hz}}), \end{aligned}$$

where $$\left| {{\tilde{\Gamma }} } \right| = 2^{\left\lfloor {\log _2 2 \cdot (C_{N_{\textrm{t}} }^1 + 4C_{N_{\textrm{t}} }^2 ) + 4C_{N_{\textrm{t}} }^2 } \right\rfloor }$$.

Based on the above analysis, this formulation demonstrates that the ESS-TTAs-MBM fundamentally enhances SE by transforming wasted spatial resources into information-carrying dimensions, paving the way for ultra-efficient MIMO-IM systems. The mathematical rigor in Eq.(12) ensures the consistency with theoretical and simulation results in the section “Simulation results”.

### Detection complexity analysis with ML algorithm

In this subsection, the detection complexities with ML detection algorithm are analyzed for different design schemes. Here, the detection complexities are measured by the real-valued multiplications.

In the proposed ESIM-MBM, according to the algorithm of the Eq. (8), the real-valued detection multiplications of the proposed ESIM-MBM with both QAM and SQAM-I are given by $C_{\mathrm{ESIM-MBM}}=8 \times N_{\mathrm r} \times 2^\eta$, where $\eta$ is the total number of transmitted bits per channel use (including AI, CI, and modulated symbol bits). This covers both single and dual active TA cases.

In the proposed ESS-TTAs-MBM, to expand the number of signal spaces, multiple types of signal constellations are employed to construct $N= 2^{\left\lfloor {{{\textrm{log}} _2 (2\times (C^1_{N\textrm{t}}+4C^2_{N\textrm{t}})+4C^2_{N\textrm{t}})}} \right\rfloor }$ number of signal vectors. Hence, its real-valued detection multiplications has three cases:

**Case 1 :** According to the Eq. (10), for the signal spaces obtained by using the first $2 \cdot (C_{N_{\textrm{t}} }^1 + 4C_{N_{\textrm{t}} }^2 )$ AI vectors in the set ${\tilde{\Gamma }}$ to modulate one QAM or SQAM-I symbol, i.e., $N \le 2 \cdot (C_{N_{\textrm{t}} }^1 + 4C_{N_{\textrm{t}} }^2 )$, satisfying the power of two. In this case, the real-valued detection multiplications for each MBM vector can be calculated as $\textrm{C}_{\mathrm{ESS - TTAs - MBM}} =8 \times N_{\textrm{r}}$.

**Case 2 :** If $2 \cdot (C_{N_{\textrm{t}} }^1 + 4C_{N_{\textrm{t}} }^2 ) \le N \le 2 \cdot (C_{N_{\textrm{t}} }^1 + 4C_{N_{\textrm{t}} }^2 ) + 2 \cdot C_{N_{\textrm{t}} }^2$, the number of transmitted components is alterable. $2 \cdot (C_{N_{\textrm{t}} }^1 + 4C_{N_{\textrm{t}} }^2 )$ of the *N* vectors are used to modulate one mapped symbol, the remaining vectors are used to modulate two mapped symbols that are from both QAM and QAM-II. In this case, when performing the operation of $\textbf{H} \cdot \textbf{X}$, the real-valued multiplication can be obtained by calculating $4N_{\textrm{r}}$ for the $2 \cdot (C_{N_{\textrm{t}} }^1 + 4C_{N_{\textrm{t}} }^2 )$ vectors being used to modulate one mapped symbol and $6N_{\textrm{r}}$ for the $2C_{N_{\textrm{t}} }^2$ vectors being used to modulate two mapped symbols. Then, performing the operation of $\left\| \cdot \right\| ^2$, the requirement of the real-valued multiplication is $4N_{\textrm{r}}$. Hence, in this case, the total real-valued multiplication for each MBM vector is

$$\textrm{C}_{\mathrm{ESS - TTAs - MBM}} = \frac{{[2 \cdot (C_{N_{\textrm{t}} }^1 + 4C_{N_{\textrm{t}} }^2 ) \cdot 8N_{\textrm{r}} + [N - 2 \cdot (C_{N_{\textrm{t}} }^1 + 4C_{N_{\textrm{t}} }^2 )] \cdot 10N_{\textrm{r}} }}{N}.$$

**Case 3 :** Similar to **Case 2**, when $2 \cdot (C_{N_{\textrm{t}} }^1 + 4C_{N_{\textrm{t}} }^2 ) + 2 \cdot C_{N_{\textrm{t}} }^2 \le N \le 2 \cdot (C_{N_{\textrm{t}} }^1 + 4C_{N_{\textrm{t}} }^2 ) + 4 \cdot C_{N_{\textrm{t}} }^2$, the number of transmitted components is also alterable. In this case, after performing the operation of $\textbf{H} \cdot \textbf{X}$ and performing the operation of $\left\| \cdot \right\| ^2$, the total real-valued multiplication for each MBM vector is

$$\textrm{C}_{\mathrm{ESS - TTAs - MBM}} = \frac{{[2 \cdot (C_{N_{\textrm{t}} }^1 + 4C_{N_{\textrm{t}} }^2 ) \cdot 8N_{\textrm{r}} + 2C_{N_{\textrm{t}} }^2 \cdot 10N_{\textrm{r}} + [N - 2 \cdot (C_{N_{\textrm{t}} }^1 + 4C_{N_{\textrm{t}} }^2 ) - 2C_{N_{\textrm{t}} }^2 ] \cdot 12N_{\textrm{r}} }}{N}.$$

Due to that these schemes such as QCM and SM-MBM only modulate one signal CP, the real-valued multiplications are the same as that of the ESIM-MBM and the ESS-TTAs-MBM with the case of $N \le 2 \cdot (C_{N_{\textrm{t}} }^1 + 4C_{N_{\textrm{t}} }^2 )$. However, for the case of $N > 2 \cdot (C_{N_{\textrm{t}} }^1 + 4C_{N_{\textrm{t}} }^2 )$, in order to achieve the extension of signal spaces, the ESS-TTAs-MBM increases the detection complexity but not much.

**Fig. 3:**
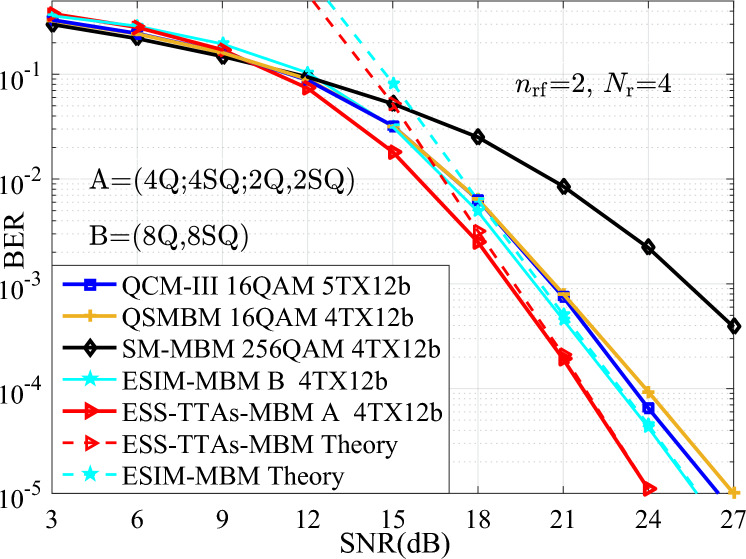
The BER performances of the proposed systems with the simulation and theoretical curves, which are compared with that of the QCM-II, QSMBM, SM-MBM systems with $\{N_{\textrm{t}},N_{\textrm{r}}\}=(4,4)$ at the SE of 12 bits/s/Hz.

**Fig. 4:**
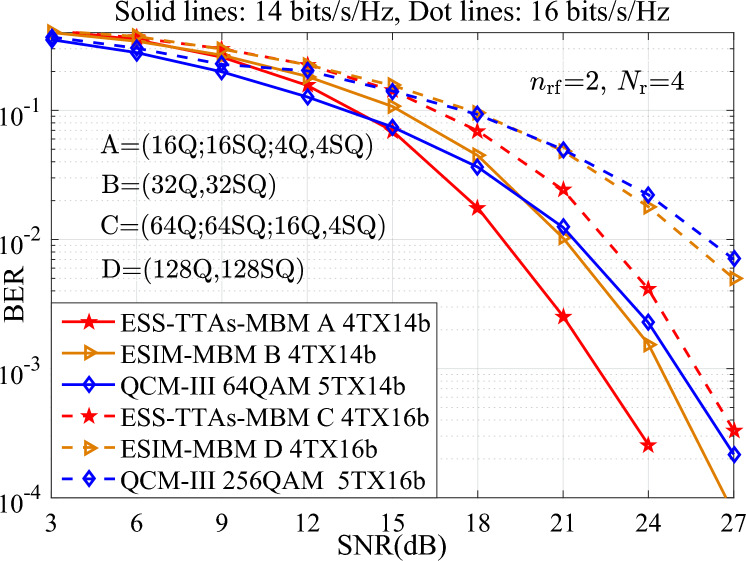
The BER performances of the proposed systems at different SEs of {14, 16} bits/s/Hz, and compared with the QCM-III with {64, 256}-QAM, $\{N_{\textrm{t}},N_{\textrm{r}}\}=(4,4)$.

**Fig. 5:**
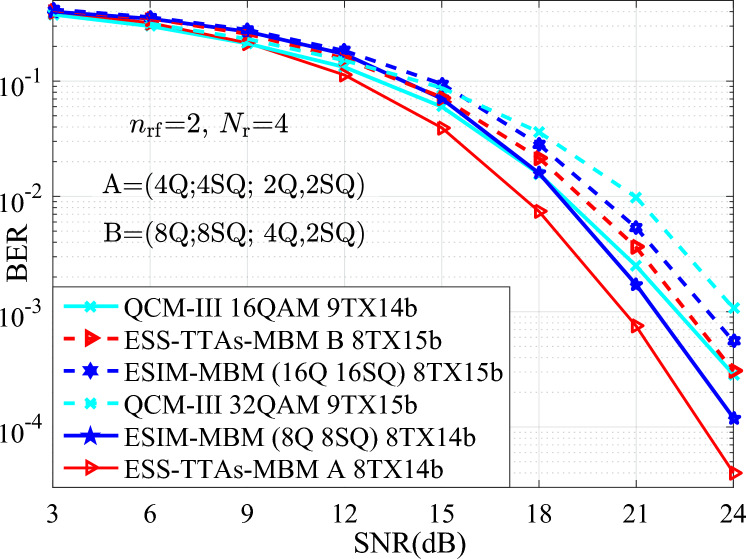
The BER comparisons between the ESS-TTAs-MBM, the ESIM-MBM and the QCM-III with $\{N_{\textrm{t}},N_{\textrm{r}}\}=(8,4)$ at the SEs of {14, 15} bits/s/Hz.

**Fig. 6:**
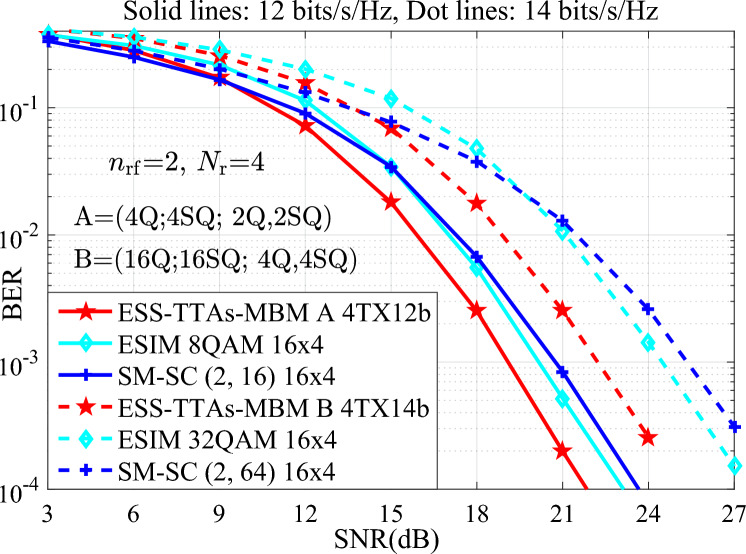
The BER performance comparisons between the ESS-TTAs-MBM and the ESIM and SM-SC systems with $N_{\textrm{r}}=4$ at the SE of 12, 14 bits/s/Hz.

In terms of time complexity, when using the same signal detector, the processing time for each detection is the same. In other words, the time complexity is mainly dominated by the detection complexity. Furthermore, the detection complexity may be quantified by the real-valued multiplications required for ML detection at the receiver. To measure the efficiency of processing the real-valued multiplications, Table 1 provides the comparisons of the real-valued multiplications for various schemes at different spectral efficiencies and transmit antennas. It can be seen from Table 1, for two cases of both $\{N_{\textrm{t}}\}=\{4, 4\}$, *N*=64, 12 bits/s/Hz and $\{N_{\textrm{t}}\}=\{8, 4\}$, *N*=256, 14 bits/s/Hz, the ESS-TTAs-MBM scheme incurs a slightly higher real-valued multiplications than the ESIM-MBM, QCM, and SM-MBM schemes. This modest increase in complexity is attributed to its enhanced design involving multiple constellation types and the exploitation of previously unused antenna index combinations to achieve higher throughput. Hence, the performance gains demonstrated in Figs. 3, 4, 5, 6 suggest this time complexity overhead is a worthwhile trade-off for the significant improvement in error performance. The real-valued multiplication counts also serve as the time complexity metric, with Table 1 providing direct cross-scheme comparisons.

**Table 1:** The complexity comparisons between the proposed schemes and the QCM and SM-MBM schemes at different spectral efficiencies and transmit antennas.

	$\{N_{\textrm{t}}\}=\{4, 4\}$, *N*=64, 12 bits/s/Hz	$\{N_{\textrm{t}}\}=\{8, 4\}$, *N*=256, 14 bits/s/Hz
ESS-TTAs-MBM	135168	532480
ESIM-MBM	131072	524288
QCM	131072	524288
SM-MBM	131072	524288

The computational load of ML detection (e.g., 532480 multiplications for 14 bps/Hz) is compatible with FPGA platforms like Xilinx RFSoC. This facilitates future real-time prototyping, though channel coherence time must exceed RF mirror switching latency to ensure CI vector reproducibility.

###  Bit error probability

In this subsection, using the ML detector at the receiver, the average BEP for the proposed ESIM-MBM and ESS-TTAs-MBM is evaluated. On the premise of the ML algorithm in Eq. (8), according to the union bounding technique^24^, the average BEP is given by

13 $$\begin{aligned} P \le \frac{1}{{2^\textbf{I} }}\sum \limits _\textbf{X} {\sum \limits _{\textbf{X} \ne {\hat{\textbf{X}}}} {\frac{{P\left( {\frac{1}{\mu } \textbf{X} \rightarrow \frac{1}{\mu }{\hat{\textbf{X}}}} \right) \cdot d\left( {\frac{1}{\mu } \textbf{X} \rightarrow \frac{1}{\mu }{\hat{\textbf{X}}}} \right) }}{I}} }, \end{aligned}$$

where ${\hat{\textbf{X}}}$ is the estimate of the detected $\textbf{X}$, $d\left( {\frac{1}{\mu } \textbf{X} \rightarrow \frac{1}{\mu }{\hat{\textbf{X}}}} \right)$ is the hamming distance between the bits corresponding to ${{\bar{\textbf{X}}}}$ and the error bits corresponding to ${\hat{ {\bar{\textbf{X}}}}}$, ${P\left( {\frac{1}{\mu } \textbf{X} \rightarrow \frac{1}{\mu }{\hat{\textbf{X}}}} \right) }$ is the unconditional pairwise error probability (PEP).

Furthermore, the unconditional PEP is obtained by taking the expectation of the conditional PEP, which is conditioned on the fading channels. According to the theory of PEP in Ref.^24^, the conditional PEP can be written by the form of the *Q*-function, as follows:

14 $$\begin{aligned} P\left( {\left. {\frac{1}{\mu } \textbf{X} \rightarrow \frac{1}{\mu }{\hat{\textbf{X}}}} \right| \textbf{H}} \right) = P\left( {\left. {\left\| {\textbf{y} - \textbf{H}\frac{1}{\mu } \textbf{X}} \right\| ^2 > \left\| {\textbf{y} - \textbf{H}\frac{1}{\mu }{\hat{\textbf{X}}}} \right\| ^2 } \right| \textbf{H}} \right) = Q\left( {\sqrt{\frac{{\left\| {\textbf{H}(\frac{1}{\mu } \textbf{X} - \frac{1}{\mu }{\hat{\textbf{X}}})} \right\| ^2 }}{{2\sigma ^2 }}} } \right) , \end{aligned}$$

Then, by the application of the alternative integral form of $Q\left( x \right) = \frac{1}{\pi }\int _0^{\frac{\pi }{2}} {\exp \left( { - \frac{{x^2 }}{{2\sin ^2 \theta }}} \right) } d\theta$, the Eq. (14) can be rewritten as

15 $$\begin{aligned} P\left( \left. {\frac{1}{\mu } \textbf{X} \rightarrow \frac{1}{\mu }{\hat{\textbf{X}}}} \right| {\textbf{H}} \right) = \frac{1}{\pi }\int _0^{\frac{\pi }{2}} {\exp \left( { - \frac{{\left\| {\textbf{H}(\frac{1}{\mu } \textbf{X} - \frac{1}{\mu }{\hat{\textbf{X}}})} \right\| ^2 }}{{4\sigma ^2 \sin ^2 \theta }}} \right) } d\theta , \end{aligned}$$

From this, taking the expectation of the Eq. (15) on the fading channel matrix $\textbf{H}$, the average PEP is given by

16 $$\begin{aligned} P\left( {\frac{1}{\mu } \textbf{X} \rightarrow \frac{1}{\mu }{\hat{\textbf{X}}}} \right) = E_{\textbf{H}} \left\{ {P\left( {\left. \frac{1}{\mu } \textbf{X} \rightarrow \frac{1}{\mu }{\hat{\textbf{X}}} \right| \textbf{H}} \right) } \right\} = \frac{1}{\pi }\int _0^{\frac{\pi }{2}} {E_\textbf{H} \left[ {\exp \left( { - \frac{{\textrm{tr}({\textbf{HDH}}^H )}}{{4\sigma ^2 \sin ^2 \theta }}} \right) } \right] } d\theta , \end{aligned}$$

where the distance matrix $\textbf{D} = \Delta \textbf{X}\Delta \textbf{X}^H ,\Delta \textbf{X} = \frac{1}{\mu }(\textbf{X} - {\hat{\textbf{X}}})$.

According to the moment generating function in Ref.^24^ and the eigenvalue decomposition of the distance matrix $\textbf{D}$, we have

17 $$\begin{aligned} E_\textbf{H} \left[ {\exp \left( { - \frac{{\mathrm{tr(} \textbf{HDH}^H )}}{{4\sigma ^2 \sin ^2 \theta }}} \right) } \right] = \det (\textbf{I} + \frac{\textbf{D}}{{4\sigma ^2 \sin ^2 \theta }})^{ - N_{\textrm{r}} } = \prod \limits _{i = 1}^r {(1 + \frac{{\lambda _i }}{{4\sigma ^2 \sin ^2 \theta }})^{ - N_{\textrm{r}} } } \end{aligned}$$

where $\lambda _i$ is the *i*-th eigenvalue of $\textbf{D} = \Delta \textbf{X}\Delta \textbf{X}^H$.

Consequently, the closed-form PEP is derived by combining the moment generating function of the Rayleigh fading channel^24^ and the eigenvalue decomposition of the distance matrix ${\textbf { D}}$, as follows:

18 $$\begin{aligned} P\left( \frac{1}{\mu } \textbf{X} \rightarrow \frac{1}{\mu }{\hat{\textbf{X}}} \right) = \frac{1}{\pi }\int _{0}^{\frac{\pi }{2}} {\mathop {\prod }\limits _{i = 1}^r {(1 + \frac{{\lambda _{i}}}{{4\sigma ^{2} \sin ^{2} \theta }})^{ - N_{\textrm{r}} } } } d\theta \end{aligned}$$

## Simulation results

In this section, to verify the advantage of the proposed ESIM-MBM with ($x_1$Q, $x_2$SQ) and the ESS-TTAs-MBM with ($x_1$Q; $x_2$SQ; $y_1$Q, $y_2$SQ), all numerical simulations of the BER versus SNR for the proposed systems are performed in MATLAB (Version R2018a, URL: *https* : //*www*.*mathworks*.*com*/*products*/*matlab*.*html*, provided under the campus-wide license of Wuzhou University), where ($x_1$Q, $x_2$SQ) denotes that $x_1$QAM or $x_2$SQAM constellation is employed for the ESIM-MBM, ($x_1$Q; $x_2$SQ; $y_1$Q, $y_2$SQ) denotes that $x_1$QAM or $x_2$SQAM constellation, or the combination of $y_1$QAM and $y_2$SQAM is employed for the ESS-TTAs-MBM, and $x_1 \times x_2=y_1 \times y_2$. In computer simulations with using the ML detector at the receiver, we set that the number of incoming data bits is 1000$\times \textbf{I}$ bits, $p_1$TX$p_2$b denotes transmitting $p_2$ bits/s/Hz using $p_1$ TAs. And, with the assumption of known channel state information at the receiver, the BER performances of the proposed systems are depicted under Rayleigh flat fading channels and compared with other systems such as the QSMBM and QCM systems in scenarios of different SEs and TAs.

In Fig. 3, the simulation and theoretical curves of the average upper BEP for the proposed ESIM-MBM and ESS-TTAs-MBM systems are depicted in scenarios of both 12 bits/s/Hz and $\{N_{\textrm{t}},N_{\textrm{r}}\}\in \{(4,4)$. It can be seen that, the simulation curves and the theoretical curves are closely overlapping at the high signal noise ratio (SNR) region. Thus, it verify the effectiveness of the proposed ESIM-MBM and ESS-TTAs-MBM. Moreover, we provide the BER performance comparison between the ESIM-MBM and ESS-TTAs-MBM systems with the QCM-III with both 16QAM and 5TX12b, the QSMBM using 16QAM, the SM-MBM using 256QAM. From the simulation results, it can be seen that, the proposed systems achieve considerably better BER performance than the classic systems, at least 0.8 SNR gains at the BER value of $10^{-3}$.

To further show the advantage of different SEs such as {14, 16} bits/s/Hz, Fig.  4 depicts the BER performances of the proposed systems and the QCM-III. From Fig.  4, compared with the QCM-III, significant SNR gains are achieved for the ESS-TTAs-MBM. For instance, 3 dB SNR gains is achieved at the BER value of $10^{-3}$. It can be seen that, the proposed systems are superior to other conventional systems under high SNR conditions, especially in the SNR region above 18 dB. This is because the ESS-TTAs-MBM further expands the size of the signal spaces and enhances the system’s anti-interference ability through dual activated TAs.

Furthermore, to further verify the performance of the proposed systems in terms of BER, more number of TAs (i.e., $N_{\textrm{t}}=8$) is considered for the simulation results of the proposed ESIM-MBM and ESS-TTAs-MBM, the QCM-III with $\{N_{\textrm{t}},N_{\textrm{r}}\}=(8,4)$ at the SEs of {14, 15} bits/s/Hz.

In Fig. 5, the BER performances of the ESIM-MBM and ESS-TTAs MBM systems are better than that of the traditional QCM-III. For example, at SNR=20 dB, the BER of QCM-III with 9TX15b is approximately $2 \times 10^{-2}$, the BER of ESIM-MBM is approximately $1 \times 10^{-2}$. the BER of ESS-TTAs-MBM is approximately $7 \times 10^{-3}$. Consequently, the proposed schemes are superior to the QCM-III, especially when SNR>20 dB. That is because, the BER performances of ESIM-MBM and ESS-TTAs-MBM, which allows for better utilization of spatial dimensions, is further improved after increasing the number of TAs, especially in the high SNR regions.

In addition, Fig. 6 with 12, 14 bits/s/Hz depicts the BER performance of the ESS-TTAs-MBM with both (4Q;4SQ; 2Q,2SQ) and (16Q;16SQ; 4Q,4SQ) in scenario of the $n_{\textrm{rf}}=2, N_{\textrm{r}}=4$, and compared with the traditional MIMO-IM systems: the ESIM with {8, 32}-ary signal constellation and 16 number of TAs, the SM-SC with both (2, 16), (2, 64)^18^ and 16 number of TAs. It can be seen that, at 12, 14 bits/s/Hz, the ESS-TTAs-MBM has significantly better than the ESIM and SM-SC in high SNR regions. For instance, at  14 bits/s/Hz and SNR=21 dB: the BER of ESS-TTAs-MBM, the ESIM and the SM-SC are approximately $2.5 \times 10^{-3}$, $1 \times 10^{-2}$, $1.5 \times 10^{-2}$, respectively. Through the above discussion, it can be seen that the ESS-TTAs MBM not only has significant performance advantages, but also significantly reduces costs and power consumption by optimizing antenna hardware design.

**Fig. 7:**
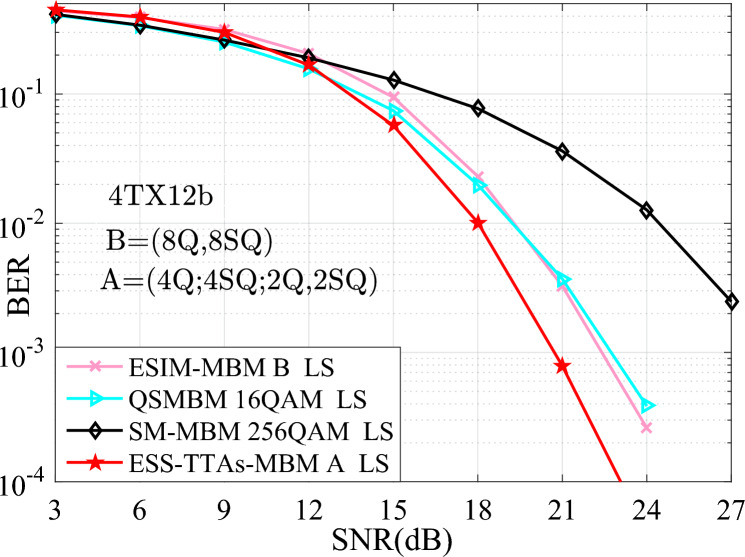
The BER comparisons of the proposed systems with the QSMBM and SM-MBM systems under the LS channel estimation in scenarios of $\{N_{\textrm{t}},N_{\textrm{r}}\}=(4,4)$ at the SE of 12 bits/s/Hz.

Additionally, in scenarios of $\{N_{\textrm{t}},N_{\textrm{r}}\}=(4,4)$ at 12 bits/s/Hz, Fig. 7 depicts the BER performance under practical Least Squares (LS) channel estimation for the proposed ESIM-MBM and ESS-TTAs-MBM systems compared to the QSMBM and SM-MBM systems, and demonstrates that the ESS-TTAs-MBM achieves the lowest BER in high SNR regions, whereas both proposed systems consistently outperform the QSMBM and SM-MBM systems in terms of BER performance. This superiority stems from the ESS-TTAs-MBM’s joint exploitation of dual-active-antenna spatial diversity and optimized constellation combinations, which effectively mitigate LS estimation errors and inter-channel interference. The results validate the robustness and practical feasibility of the proposed designs in real-world imperfect CSI scenarios.

## Conclusions

In this article, on the premise of the variability between one active TA and two active TAs, two research works of the ESIM-MBM and the ESS-TTAs-MBM, which can enhance the SE and BER performances, have been investigated. Firstly, the deep integration of the MIMO-IM system and the MBM, called as ESIM-MBM, is designed for improving the SE by developing the space and channel domains. Furthermore, by utilizing the combination design of multiple signal constellations, the ESS-TTAs-MBM has been proposed to further expand the number of signal spaces for the exploitation of the additional information, along with the channel domain. In addition, by analyzing the design of signal spaces in the ESIM, the design method of expanding the signal spaces has been proposed. Finally, on the basis of the ML detector at the receiver, the computational complexity with the real multiplication are presented for the different design schemes, and the average BEP is also presented. Simulation results show that, the BER versus SNR performances of the proposed schemes outperforms that of different schemes such as QSMBM, QCM, SM-MBM in the scenarios of different SEs. Future work will implement an FPGA-based prototype (e.g., Xilinx RFSoC) using reconfigurable antennas^2^ to validate real-time performance under mobility and hardware non-idealities.

## Data Availability

The datasets used and/or analysed during the current study available from the corresponding author on reasonable request.
